# Prevalence and solving strategies of drug-related problems in adult psychiatric inpatients - a systematic review

**DOI:** 10.3389/fpsyt.2024.1460098

**Published:** 2024-12-04

**Authors:** Katharina Wien, Pamela Reißner, Gudrun Hefner, Julia Thern, Stefan Borgwardt

**Affiliations:** ^1^ Hospital Pharmacy, University Medical Center Schleswig-Holstein, Lübeck, Germany; ^2^ Department of Psychiatry and Psychotherapy, Center for Integrative Psychiatry, University Medical Center Schleswig-Holstein, Lübeck, Germany; ^3^ Department of Psychiatry and Psychotherapy, Vitos Klinikum Hochtaunus, Bad Homburg, Germany; ^4^ Psychiatric Hospital, Vitos Clinic for Forensic Psychiatry, Eltville, Germany; ^5^ Department of Psychiatry and Psychotherapy, Center of Brain, Behavior and Metabolism, University of Lübeck, Lübeck, Germany

**Keywords:** drug-related problems, psychiatry, mental health, inpatient, clinical interventions, psychopharmacotherapy, clinical pharmacists, interdisciplinary collaboration

## Abstract

**Introduction:**

Most psychiatric inpatients receive psychopharmacological treatment indicated for their mental diseases. The aim of this systematic review is to give clinical pharmacists and physicians a comprehensive summary of common drug-related problems (DRPs) in adult psychiatric inpatients and of potential interventions to solve them in clinical practice.

**Methods:**

Six databases and registers were searched for English, German and French articles published between 1999 and 2023 with content regarding the prevalence and/or type or interventions to solve DRPs in adult psychiatric inpatients. Studies were categorized based on types of DRPs and clinical interventions. The prevalence rates of DRPs and subtypes were compared quantitatively and the tested interventions were summarized qualitatively.

**Results:**

A total of 88 articles with an overall sample of over 95.425 adult psychiatric inpatients were included in this review. DRPs were reported with a prevalence range of 0.32 to 9.48 per patient. The most frequently reported DRPs were caused by prescribing errors (1.91 per patient), the most frequent subtype was drug interaction (0.77 per patient). Clinical pharmacists were involved in interventions in 7 of 13 included articles. Interventions consisted of clinical pharmacy services on the ward, educational classes, medication reviews, and the implementation of digital tools such as dispensing cabinets and prescribing tools.

**Discussion:**

The included studies were heterogeneous. The most frequent DRPs in psychiatry are related to prescribing errors and drug interactions. Clinical pharmacists may support the drug therapy by identifying and effectively solving DRPs in psychiatric inpatients using interdisciplinary approaches.

**Systematic review registration:**

https://www.crd.york.ac.uk/prospero/, identifier CRD42022354958.

## 1 Introduction

Since the American Institute of Medicine published their report “To Err is Human: Building a Safer Health System” in 1999 (1), medication errors have received more attention in clinical research. A medication error (ME) is defined as “an unintended failure in the drug treatment process that leads to, or has the potential to lead to, harm to the patient” (2). Medication safety is an important part of patient safety. However, in a comprehensive systematic review on “Patient safety in inpatient mental health settings”, Thibaut et al. (3) found only 17 articles related to medication safety, including five studies on adverse drug events (4–7). An adverse drug event (ADEs) is defined as “any untoward medical occurrence in a patient or clinical trial subject administered a medicinal product [ … ] which does not necessarily have a causal relationship with this treatment” (2). In contrast to an ADE, an adverse drug reaction (ADR) is defined as “an appreciably harmful or unpleasant reaction, resulting from an intervention related to the use of a medicinal product” (8).

Previously, in 2003, Grasso et al. found few reports on the incidence and characteristics of MEs in psychiatric hospitals (9). In the following years, further reviews on MEs have been published (10–12). In the most recent systematic review on MEs and ADEs in both inpatient and outpatient settings of mental health hospitals, 20 articles were identified and MEs and ADEs were categorized as prescribing errors (PEs), unintentional medication discrepancies, transcription errors, medication administration errors (MAEs), and dispensing errors (12) with an overall ME rate of 10.6–17.5 per 1000 patient-days (13, 14), 17.4% of total opportunities for error (4) and in 61.4% of patients (15). MEs and ADEs are categories of drug-related problems (DRPs) which are defined as “an event or circumstance involving drug therapy that actually or potentially interferes with desired health outcomes” (16). In the past six years since the last review’s publication, more reports on a broader range of DRPs have emerged, including pharmacodynamic and pharmacokinetic drug-drug interactions (17, 18), potentially inappropriate prescribing in older psychiatric patients (19), polypharmacy in psychiatry (20), prevalence, nature, severity and preventability of ADEs (21), and on clinical pharmacist interventions to solve DRPs (22).

Pharmacotherapy is an important part of the treatment of psychiatric patients, especially of those treated in hospitals due to the acute severity of their diseases. Psychiatric patients often need to take their prescribed psychotropic drugs for a longer time. Therefore, it is crucial for clinicians to be aware of the most prevalent DRPs occurring in psychiatric inpatients and to implement effective interventions to prevent or solve these DRPs before patients are discharged to ambulatory care. One possible way to identify medication discrepancies at transitions of care is medication reconciliation at hospital admission and discharge which is a process usually completed by clinical pharmacists (23). Its positive impact has been shown in a mental health hospital (24).

An important base for designing effective clinical interventions is the knowledge of potential risk factors for DRPs. A systematic review published in 2022 focused on risk factors for DRPs in hospital-based mental health units (25). The authors identified an increasing number of prescribed medications as the only factor consistently reported to be significantly associated with the occurrence of most types of DRPs in eleven of 14 included articles (25).

Furthermore, it has been established that many ADRs occur dose-dependently and therefore depending on drug blood concentrations (26). Clinical guidelines on psychopharmacological treatment provide recommendations for therapeutic drug monitoring (TDM) including therapeutic reference ranges in blood concentrations of many drugs (26). In Germany, the Consensus Guidelines for Therapeutic Drug Monitoring in Neuropsychopharmacology are well established for the interpretation of psychotropic drug concentrations in blood (26). Results from clinical studies which assessed blood concentrations of drugs with regard to recommended therapeutic reference ranges in patients experiencing DRPs, especially ADRs, would be helpful to guide future dosing decisions in clinical practice.

A number of reviews on different aspects of medication safety in psychiatric settings, e.g. medication safety in mental health in inpatient and outpatient settings (27), MEs and ADEs in mental health hospitals (12), MEs in older people with mental health problems (28), the prevalence and characteristics of psychotropic-related hospitalizations in older people (29), neuroleptic malignant syndrome (30), and certain interventions for its improvement in psychiatric settings, such as text messaging interventions to promote medication adherence (31) and clinical pharmacist interventions (22), have been published over the past twenty years.

However, no systematic review has yet been published on the overall prevalence of DRPs in the psychiatric inpatient setting and interventions to solve them.

With this systematic review, we aim to give an up-to-date overview to clinicians on the existing literature on a broad range of DRPs and interventions to solve them in the psychiatric inpatient setting. This review addresses the following questions: What are the most frequent DRPs and DRP subtypes in adult psychiatric inpatients and which interventions have been tested to solve them?

## 2 Methods

The protocol for this systematic review was prepared according to the Preferred Reporting Items for Systematic Reviews and Meta-Analyses (PRISMA) 2020 checklist (32) and was registered with PROSPERO (CRD42022354958 (33),).

The definitions used in this review are given in **Table 1**.

**Table 1:** Definitions used in this review.

Term	Definition
Drug-Related Problem (DRP)	The Pharmaceutical Care Network Europe (PCNE) defines a DRP as “an event or circumstance involving drug therapy that actually or potentially interferes with desired health outcomes” (16). DRPs investigated in this study included adverse drug events, adverse drug reactions, medication errors, drug interactions, risky drug combinations, contraindications, and medication nonadherence. Medication nonadherence was added as an investigated DRP type after initial registration of the study protocol.
Medication Error (ME)	In the “Good practice guide on recording, coding, reporting and assessment of medication errors”, the European Medicines Agency defined a ME as “an unintended failure in the drug treatment process that leads to, or has the potential to lead to, harm to the patient” (2).
Adverse Drug Event (ADE)	ADEs are defined as “any untoward medical occurrence in a patient or clinical trial subject administered a medicinal product [ … ] which does not necessarily have a causal relationship with this treatment” (2). Those associated with medication errors are defined as preventable ADEs (pADE) (12).
Adverse Drug Reaction (ADR)	An ADR is defined as “an appreciably harmful or unpleasant reaction, resulting from an intervention related to the use of a medicinal product, which predicts hazard from future administration and warrants prevention or specific treatment, or alteration of the dosage regimen, or withdrawal of the product” (8).
Drug interaction	A drug interaction is defined as any combination of two or more agents which could adversely change the efficacy or tolerability of each other (34). All types of drug interactions including pharmacokinetic drug-drug interactions (pkDDIs), pharmacodynamic drug-drug interactions (pdDDIs), drug-disease interactions and drug-food interactions were included.
Contraindication	“A contraindication is a circumstance, condition, symptom, or factor that increases the risk associated with a [ … ] drug [ … ]. A contraindication refers to any intervention considered inappropriate or inadvisable based upon unique factors of the situation such as potential harmful interactions between drugs or medical conditions that renders an individual vulnerable if implemented (35)”.
Medication nonadherence	Medication nonadherence is defined as the extent to which patients are unable to follow the recommendations for prescribed treatments including taking a different dose than prescribed, taking prescribed drugs at the wrong time or not taking prescribed drugs at all (36).
Potentially inappropriate medication (PIM)	Drugs which may cause significant harm in older patients ≥65 years are referred to as potentially inappropriate medication (PIM) (19). Different lists of PIMs containing a multitude of drugs have been developed (37–39).
Medication review	Medication review is a structured evaluation of a patient’s medicines with the aim of optimizing medicines use and improving health outcomes. This entails detecting DRPs and recommending interventions (40).
Medication reconciliation	Medication reconciliation is an activity or a combination of activities to get a correct and total picture of the intended (and real) drug use of a patient at all care-transitions without necessarily detecting DRPs other than non-intended medication discrepancies. It may therefore be a first part of medication review (40).

### 2.1 Search strategy

A search strategy was developed using the advanced search algorithms on the six databases listed below. The keywords were searched in titles and abstracts of articles. The search strategy included seven main keywords for DRPs (drug related problems, adverse drug events, medication errors, adverse drug reactions, drug interactions, contraindications and combination) and nine keywords for the study population and setting (psychiatry, mental health, inpatients, hospital, tertiary care, day hospital; NOT pediatric, children, adolescent). For the full search strategies, see the **Supplementary Material**. All types of studies published in English, German or French language between 1 January, 1999 and 31 December, 2023 were included. The language restriction was chosen because most relevant articles were expected to be published in these languages. The year 1999 was chosen as it was the year the report “To err is human” was written (1) and 1999 and 2000 were the years commonly used as starting dates for similar literature reviews (12, 25). The search strategy was tested by one author (KW) and discussed with two further authors (PR, GH) before the start of the main search.

### 2.2 Information sources

The following databases were searched in October 2022: MEDLINE via PubMed, Scopus, Google Scholar, The Cochrane Library, including the Cochrane Database of Systematic Reviews, PROSPERO, and clinicaltrials.gov. An alert was created on PubMed, Scopus and the Cochrane Library to receive new search results for the saved searches weekly via e-mail until December 2023. In addition to the protocol-driven search, the reference lists of the studies included in the review were checked manually for any relevant studies not identified by the computerized literature search and further relevant articles personally known by the study authors were checked against eligibility criteria (41).

### 2.3 Eligibility criteria

#### 2.3.1 Inclusion criteria

All study types (including reviews and meta-analyses) were included, e.g. qualitative studies and surveys, prospective studies, retrospective studies, and case reports, that investigated a case or the prevalence of DRPs in psychiatric inpatients or patients in day hospital care and/or potential interventions aiming to solve them as primary or secondary outcomes if they included adult patients older than 18 years and if they were published between 1 January 1999 and 31 December 2023 (instead of 31 October 2022 as originally planned).

Both randomized and non-randomized interventional studies were included, as it is difficult to randomize groups when observing and analyzing DRPs. Most relevant studies were expected to have used a non-randomized study design. Since randomized controlled trials produce a higher level of evidence, studies were also included if they used a randomized design.

Multiple different classification systems for DRPs have been reported and translated to different languages for clinical use, e.g. “The PCNE Classification” for DRPs (42) and the NCC MERP Taxonomy of Medication Errors (43). Regardless of the classification system used, all reported DRPs from studies meeting inclusion criteria were included in the review.

After initial registration of the study protocol, it was specified that case reports on DRPs, especially ADRs caused by MEs, studies conducted in day hospital care and articles reporting either DRP prevalence rates or interventions to solve them, but not necessarily both, would be included in this review.

#### 2.3.2 Exclusion criteria

Studies were excluded if full-text articles were not available, if they were conducted in general hospitals and data from the psychiatric department could not be extracted, if the methodology used to identify DRPs was not sufficiently described, if they reported ADR only for a specific drug or drug group without assessing an intervention for their prevention, and if statistical testing to evaluate their conducted interventions to solve DRPs was not performed. Commentaries, editorials, viewpoint articles, letters, and further additionally added article types after registration of the study protocol (books, study protocols of uncompleted studies, phase I or II clinical trials, poster and conference abstracts) were also excluded.

During full text screening, it was decided that all articles which reported the prevalence of potentially inappropriate medication (PIM) in elderly patients without any other DRPs such as ADRs as defined in our inclusion criteria were excluded. This decision was based on the fact that there is not a gold standard for content-related appropriate medication prescriptions and PIM does not necessarily have to lead to a manifest DRP in a patient prescribed with one of these drugs.

### 2.4 Selection process

Titles and abstracts identified in the computerized searches on the six databases were screened for eligibility by one author (KW). Two authors (PR, GH) approved the screening based on a random of 10% of the studies (290 of 2827 articles), a good agreement (≥ 80%) of 84.8% (246/290 articles) was achieved. Included studies were approved by three authors (KW, PR, GH). In case of disagreement, studies were discussed and deliberated about whether all inclusion and exclusion criteria were met. In case of uncertainty, the articles were retained for full text screening.

### 2.5 Data collection process and data items

Two separate data extraction forms were developed to collect data from original studies and from (systematic) reviews, respectively. To ensure that the data extraction forms were comprehensive and that the data collection process was reliable, data extraction was performed independently for 20 articles during full text screening by three authors (KW, PR, GH). After achieving a good agreement of more than 80%, data from the remaining full texts was extracted by one author (KW) to save time resources (44). Types of DRPs were categorized according to categories listed in the guideline for medication management in pharmacies by the German Chamber of Pharmacists (45). Further categories were added based on the PCNE classification of DRPs V9.1 (46). All DRP classification categories used in this review with their corresponding definitions are listed in **Table 2**. The following data was collected from original studies: Title, authors, country, year of publication, demographics, aim and objectives of the study, study setting, study design, duration of the study, sample size, inclusion and exclusion criteria, data collection method, data collectors, type of prescription process (paper/electronic charts), DRPs identification method, types, subtypes and rates of DRPs investigated, drugs responsible for DRPs, number of patients with DRPs, total number of DRPs, severity of reported errors, if blood concentration of drugs was analyzed and correlated with DRPs (especially ADRs), if applicable: description of intervention to solve DRPs, unsolved DRPs after the intervention, statistical methods, and funding sources. The following data was additionally collected from (systematic) reviews: databases used for literature search, overall rates of DRPs.

**Table 2:** Drug-related problem (DRP) classification categories and their definitions based on the guideline for medication management by the German Chamber of Pharmacists (BAK) (45, 47) and the PCNE classification of DRPs V9.1 (46).

DRP category	DRP definition	Source
Problem: Treatment effectiveness	There is a (potential) problem with the (lack of) effect of the pharmacotherapy.	PCNE V9.1 P1
Problem: Treatment safety	Patient suffers, or could suffer, from an adverse drug event.	PCNE V9.1 P2
Adverse drug reactions/drug intolerance	Adverse drug event (possibly) occurring.	BAK, PCNE V9.1 P2.1
Causes		
(Pseudo-) Double medication	Inappropriate duplication of therapeutic group or active ingredient.	BAK, PCNE V9.1 C1.4
Interactions (drug-drug, drug-food, drug-alcohol, drug-tobacco, drug-disease)	Inappropriate combination of drugs, or drugs and herbal medications, or drugs and dietary supplements, or drugs and alcohol, or drugs and tobacco, or drugs in preexisting diseases.	BAK, PCNE V9.1 C1.3
Drug selection inadequate	Inappropriate drug according to guidelines/formulary, no indication for drug, no or incomplete drug treatment in spite of existing indication or too many different drugs/active ingredients prescribed for indication (excluding (pseudo-) double medication and drug interactions).	PCNE V9.1 C1.1-2, C1.5-6
Dose selection inadequate	Drug dose too low or too high, dosage regimen not frequent enough or too frequent.	BAK, PCNE V9.1 C3.1-4
Time of intake inadequate	Dose timing instructions wrong, unclear or missing.	BAK, PCNE V9.1 C3.5
Treatment duration	The duration of treatment is too short or too long.	PCNE V9.1 C4
Dosage form inadequate	The cause of the DRP is related to the selection of the drug form (inappropriate drug form/formulation).	BAK, PCNE V9.1 C2
Patient-related drug use problems	The cause of the DRP is related to the patient and his behavior (intentional or non-intentional): Patient takes food that interacts, uses inappropriate timing or dosing intervals, unintentionally administers/uses the drug in a wrong way, is physically unable to use drug/form as directed or is unable to understand instructions properly.	BAK, PCNE V9.1 C7.5, C7.7-10
Storage inadequate	Patient stores drug inappropriately (e.g. at wrong temperature, outside of primary package).	BAK, PCNE V9.1 C7.6
Non-adherence/Non-compliance	Patient intentionally uses/takes less or more drug than prescribed or does not take the drug at all for whatever reason, Patient unintentionally administers/uses the drug in a wrong way	BAK, PCNE V9.1 C7.1-2, C7.8
Dispensing	The cause of the DRP is related to the logistics of the prescribing and dispensing process.	PCNE V9.1 C5
Administration (Drug use)	The cause of the DRP is related to the way the patient gets the drug administered by a health professional or other carer, despite proper dosage instructions (on label/list)	PCNE V9.1 C6
Patient transfer related (Medication reconciliation)	The cause of the DRP can be related to the transfer of patients between primary, secondary and tertiary care, or transfer within one care institution.	PCNE V9.1 C8.1
Other causes	No or inappropriate outcome monitoring (incl. TDM), other causes or no obvious cause.	PCNE V9.1 C9
Problems with self-medication	Patient has problems with medication used as self-medication which has not been prescribed by a physician.	BAK
Indication inadequate for self-medication	The indication must not be treated as self-medication and a physician must be consulted.	BAK
Product in self-medication inadequate for indication	Self-medication is possible but the over-the-counter drug used by the patient is inadequate for the present indication.	BAK
Drug dose in self-medication too low or too high	Drug dose is subtherapeutic or in toxic range.	BAK
Contraindication in self-medication	Drug is inadequate for patient, e.g. due to diseases, allergies or age.	BAK

TDM, Therapeutic Drug Monitoring.

### 2.6 Quality and bias assessments

Quality and bias of included studies were assessed by one author (KW).

#### 2.6.1 DRP reporting quality assessment

Quality assessment of the individual studies regarding reporting of DRPs was based on the criteria established by Allan and Barker (1990) (48), which have previously been used for other systematic reviews on MEs (12, 49, 50). A maximum of 12 points corresponding to high quality could be reached (**Table 3**).

**Table 3:** Criteria for the quality assessment of included studies based on the criteria established by Allan and Barker (48).

Criterion	Points
Aims/objectives of the study clearly stated.	1
Definition of what constitutes a DRP/preventable DRP.	1
DRP/preventable DRP categories specified • categories specified, no validated classification system used or named • categories specified, validated classification system used (e.g. PCNE, NCC MERP, Pi-Doc).	1 2
DRP/preventable DRP categories defined.	1
Presence of a clearly defined denominator (e.g. percentage rate of DRPs as number of actual DRPs divided by number of prescriptions/patients or rate of DRPs per 1000 patient days).	1
Data collection method described clearly (e.g. direct observation, incident reports, chart review).	1
Study setting described.	1
Validity measure in place to confirm the occurrence of DRP/preventable DRP • observation by a pharmacist or senior clinical pharmacology physician • validity/causality scale used (e.g. Naranjo algorithm- ADR probability scale, the UKU side effect rating scale).	1 2
Reliability measures (e.g. inter-rater reliability expressed by Cohen’s kappa, Fleiss’s kappa or Krippendorff’s alpha).	1
Study limitations listed.	1
**Maximum score**	**12**

DRP, Drug-related problem; ADR, Adverse drug reaction.

Due to the heterogeneity of DRPs and since no validity measure for the detection of DRPs has been defined as gold standard, the observation of DRPs by a pharmacist or by a senior clinical pharmacology physician (1 point in the quality assessment) was considered as a poorer validity measure than the use of a validated validity or causality scale (2 points in the quality assessment; e.g. Naranjo algorithm-ADR probability scale, UKU side effect rating scale). To rule out the subjectivity of detection of DRPs, a study on DRPs with high quality should have assessed inter-rater reliability by calculating a reliability coefficient.

#### 2.6.2 Study type specific quality assessment

The selected studies were assessed for bias by the applicable JBI critical appraisal checklists by the University of Adelaide, available at https://jbi.global/critical-appraisal-tools (51). A total score achieved out of all study type specific criteria was calculated. The risk of bias was ranked according to the JBI criteria with ≤ 39% as high, 40% to 69% as moderate and ≥70% as low risk of bias.

#### 2.6.3 Risk of bias assessment

An assessment of meta-biases such as publication bias across studies and selective reporting within studies was completed. To achieve a high standard of reporting the updated ‘Preferred Reporting Items for Systematic Reviews and Meta-Analyses’ (PRISMA) 2020 statement (32) was adopted. Before publication, the interventional studies included in this review were assessed for bias by the AMSTAR 2 tool (44).

#### 2.6.4 Certainty assessment

A final grading of available evidence was included in a summary of findings table of studies reporting the prevalence of DRPs or MEs using the Grading of Recommendations, Assessment, Development and Evaluation (GRADE) system (52). Interventions assessed in the included articles were not comparable and therefore not included in the summary of findings table.

### 2.7 Data synthesis and analysis

#### 2.7.1 Strategy for data synthesis

Studies were aggregated based on classification of DRPs and conducted interventions. A distinction was made between manifest errors with or without an ADR, intercepted errors and potential errors with regard to preventability of errors. If study results appeared to be heterogenous in nature, underlying causes were investigated.

#### 2.7.2 Effect measures

The main outcomes of this review were the prevalence and types of DRPs and tested interventions. The prevalence was extracted from the included studies as the number of DRPs per 100 patients or per 1000 patient-days or per 100 opportunities for error. As an additional outcome, the percentages or rates of unsolved DRPs after an intervention were included from interventional studies. Furthermore, the included articles were checked for the availability of correlations between blood concentration of drugs and prevalence of DRPs, especially ADRs.

#### 2.7.3 Data analysis

The included study data were inconsistent as different methods for detection, classification and reporting of DRPs were used. Therefore, a quantitative meta-analysis of the prevalence rates was not possible. Instead, results were summarized separately for each DRP category. The rate of DRPs, ADEs and MEs was usually calculated as a percentage rate of all patients/prescriptions or as a rate of DRPs, ADEs or MEs per 1000 patient days. The percentage rate of DRPs was calculated by dividing the number of actual DRPs that occurred or number of patients or prescriptions affected by DRPs by the total number of prescriptions or patients multiplied by 100. The rate of DRPs per 1000 patient days was determined by dividing the number of DRPs by the total number of patient-days multiplied by 1000.

### 2.8 Analysis of subgroups or subsets

When DRPs were not generally reported in a study, the prevalence rate per 100 patients or rate of a specific DRP (e.g. ADE, drug-drug interaction (DDI)) per 1000 patient-days was calculated. A subgroup analysis for DRPs in patients ≤ 65 years or > 65 years was planned but not calculated as few studies reported corresponding data.

## 3 Results

**Figure 1:**
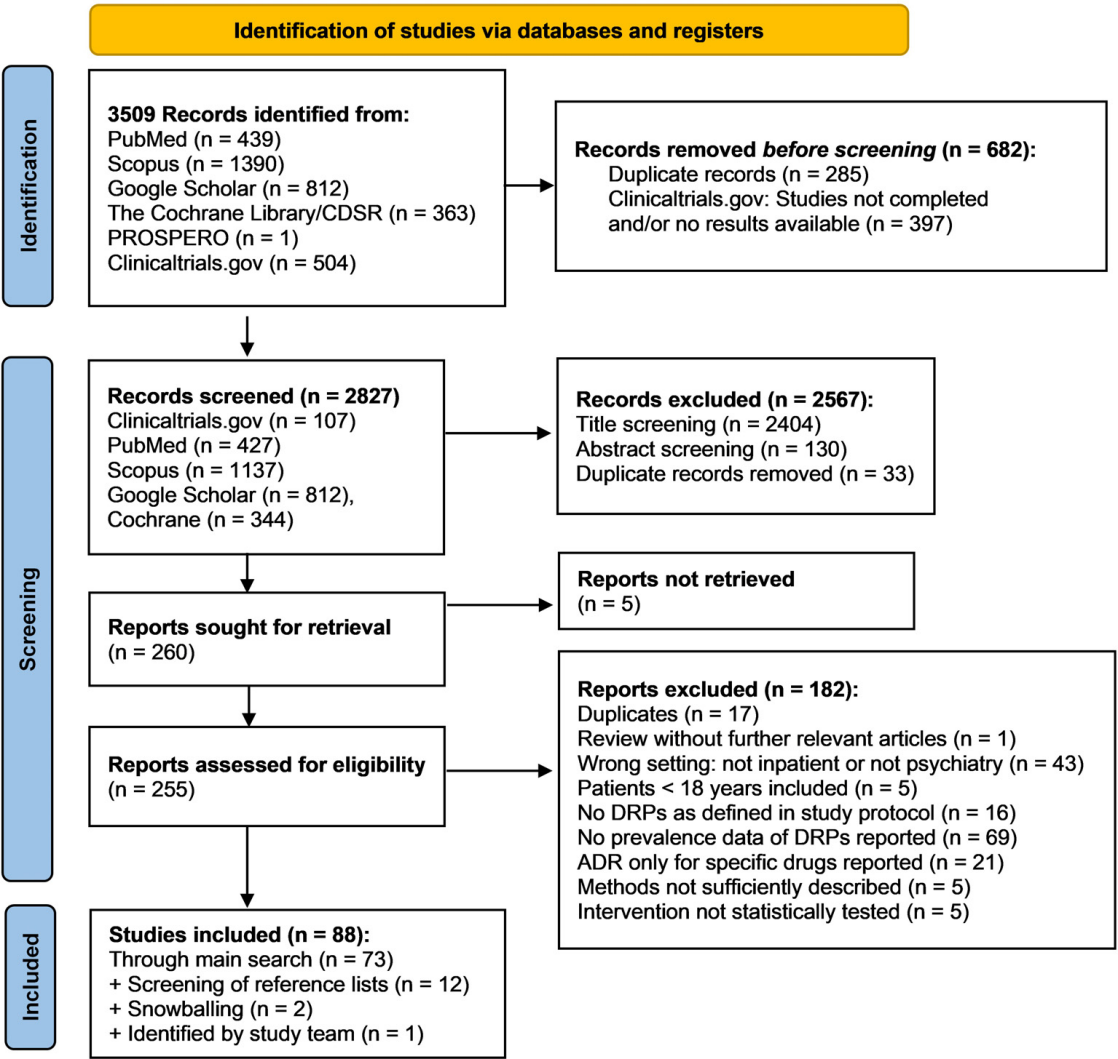
PRISMA flow diagram (32) of the search and selection process of studies for inclusion in the review.

In the main literature search, 3509 records were identified from the databases. Among the total of 255 reports assessed for eligibility during full-text screening, 182 did not meet inclusion criteria and were therefore excluded. The remaining 73 studies were included in the review. By screening the reference lists of included studies and of excluded reviews, 12 additional studies were identified. Two further studies were identified using a snowballing technique based on the most relevant studies on DRPs retrieved in the main search. Lastly, one additional recently published study meeting inclusion criteria came to the attention of the study team. Overall, 88 studies were included in the review. The details of the search and selection process are presented in a PRISMA flow diagram (**Figure 1**). A list of excluded studies assessed for eligibility by full text screening and the respective justifications for exclusion is available online in the **Supplementary Material** as an Excel sheet.

### 3.1 Characteristics of included studies

Among the 88 articles included in the review, 39 were prospective, observational studies (7, 13, 20, 53–88), 30 were retrospective studies (21, 89–117), two were mixed-methods studies (4, 118), and four were case series or case reports (119–122). 13 interventional studies were identified, eleven of them used a prospective (123–133) and two a retrospective design (134, 135). No review identified in the literature search met inclusion criteria as none was conducted only in inpatient settings. Overall, DRPs identified in 95.425 adult subjects (45.7% female, if reported) and by incident reports based on 192.372 admissions were included. In six reports, the number of subjects or admissions was not reported. Data synthesis of demographic characteristics was not possible due to the methodological heterogeneity of the studies. For each individual study, demographic details are shown in the data extraction form in the **Supplementary Material**.

**Figure 2:**
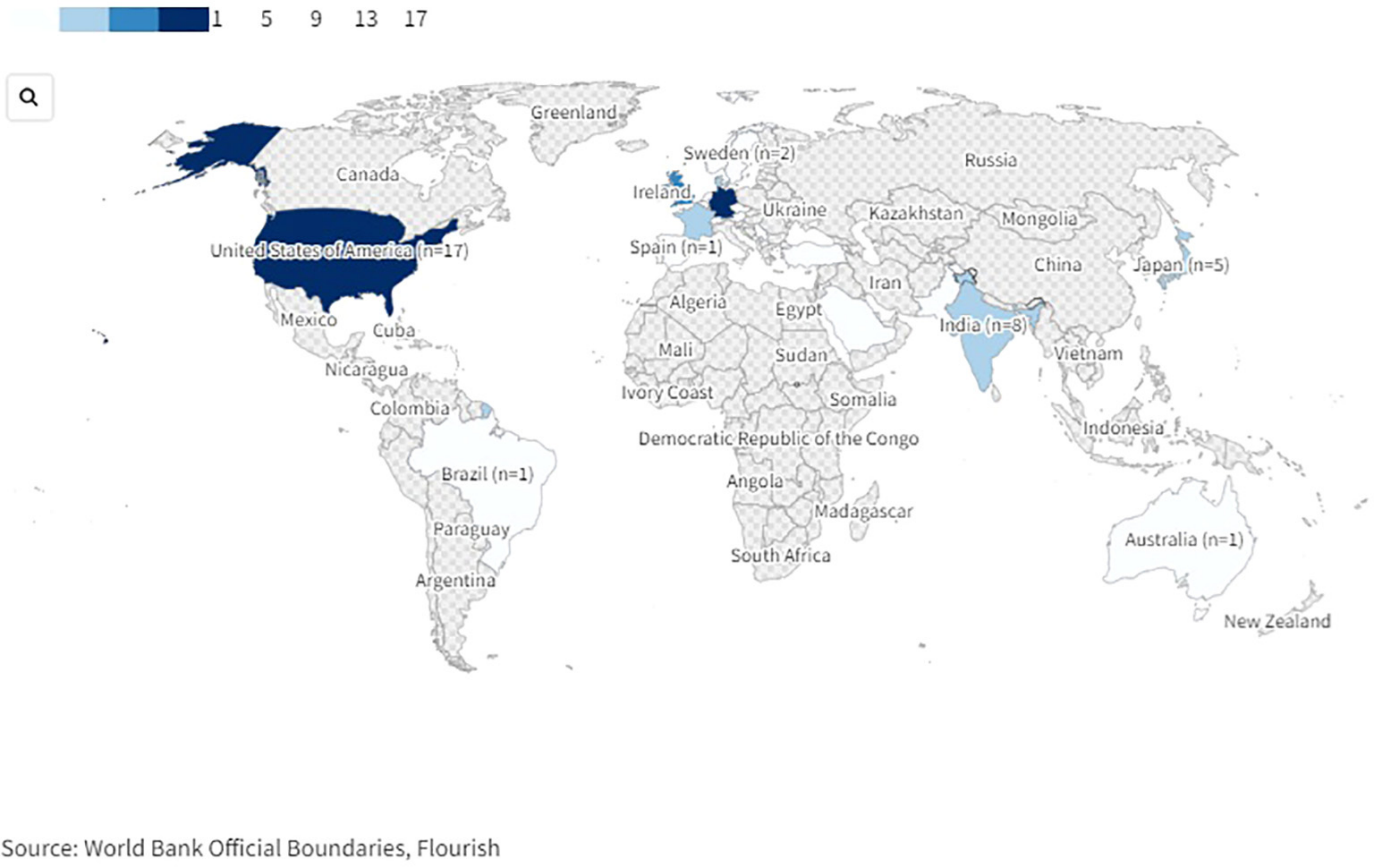
World map of countries in which included studies on drug-related problems in psychiatric inpatients were conducted. An interactive version of this projection map is available online: https://public.flourish.studio/visualisation/17489814/. Created with flourish.studio (https://flourish.studio).

The studies were conducted in various countries world-wide (**Figure 2**). 53 studies were conducted in Europe: 14 in Germany (61, 63, 66, 71, 90, 94, 96, 97, 105, 109, 122, 123, 128, 134), eleven in the UK (21, 53, 54, 69, 75, 80, 83–85, 101, 124), six in France (55, 60, 76, 79, 89, 114), five in Denmark (4, 86, 103, 113, 131), two each in Belgium (58, 59), Sweden (64, 99), The Netherlands (70, 104), and Turkey (92, 133), one study each in Austria (67), the Czech Republic (121), Montenegro (130), Norway (62), Portugal (68), Serbia (81), Spain (7), and Switzerland (20), and one multicentric study was conducted in Germany and Switzerland (95). 17 studies were conducted in the USA (13, 56, 65, 78, 91, 93, 100, 106, 107, 115, 118–120, 125–127, 135). Eight further studies were conducted in India (72–74, 77, 82, 88, 110, 111), five in Japan (57, 98, 102, 129, 132), two in Pakistan (108, 117), and one study each in Australia (112), Brazil (116) and Saudi Arabia (87).

Eight of the included studies reported the prevalence of DRPs in general (72, 87, 88, 96, 103, 110, 123, 130) and included a total of 2.208 psychiatric inpatients. Five of these studies described the results of interventions to solve DRPs (87, 88, 103, 123, 130), the other three were non-interventional studies (72, 96, 110). None of these studies reported blood concentrations of drugs involved in DRPs. Two further studies reported the prevalence of different types of DRPs including drug interactions, contraindications, and prescription errors (PEs) without aiming to include DRPs in general (63, 105).

The remaining 78 studies assessed the prevalence or cases of specific types of DRPs such as ADEs, ADRs, drug-drug interactions or prescription errors. 36 studies reported ADEs or ADRs, some among other types of DRPs (7, 20, 21, 56, 61, 62, 64, 66, 67, 70, 71, 73, 74, 77, 89, 91–93, 95, 98–100, 102–104, 107, 109, 113, 119, 120, 122, 125, 126, 128, 133, 134). 24 articles assessed the prevalence of drug interactions (54, 56, 58, 59, 76, 77, 81–83, 91, 92, 94, 97, 101, 108, 111, 112, 115–117, 121, 122, 128, 134). 26 studies reported on MEs; seven of them in general (4, 13, 57, 78, 102, 107, 118), five of them on MAEs (53, 80, 85, 93, 124) and 14 articles focused on PEs including transfer-related PEs (55, 60, 65, 68–70, 75, 79, 84, 86, 106, 114, 131, 135). Lastly, 5 studies assessed medication adherence or medication (non-) compliance as a subtype of DRPs (90, 127–129, 133).

**Figure 3:**
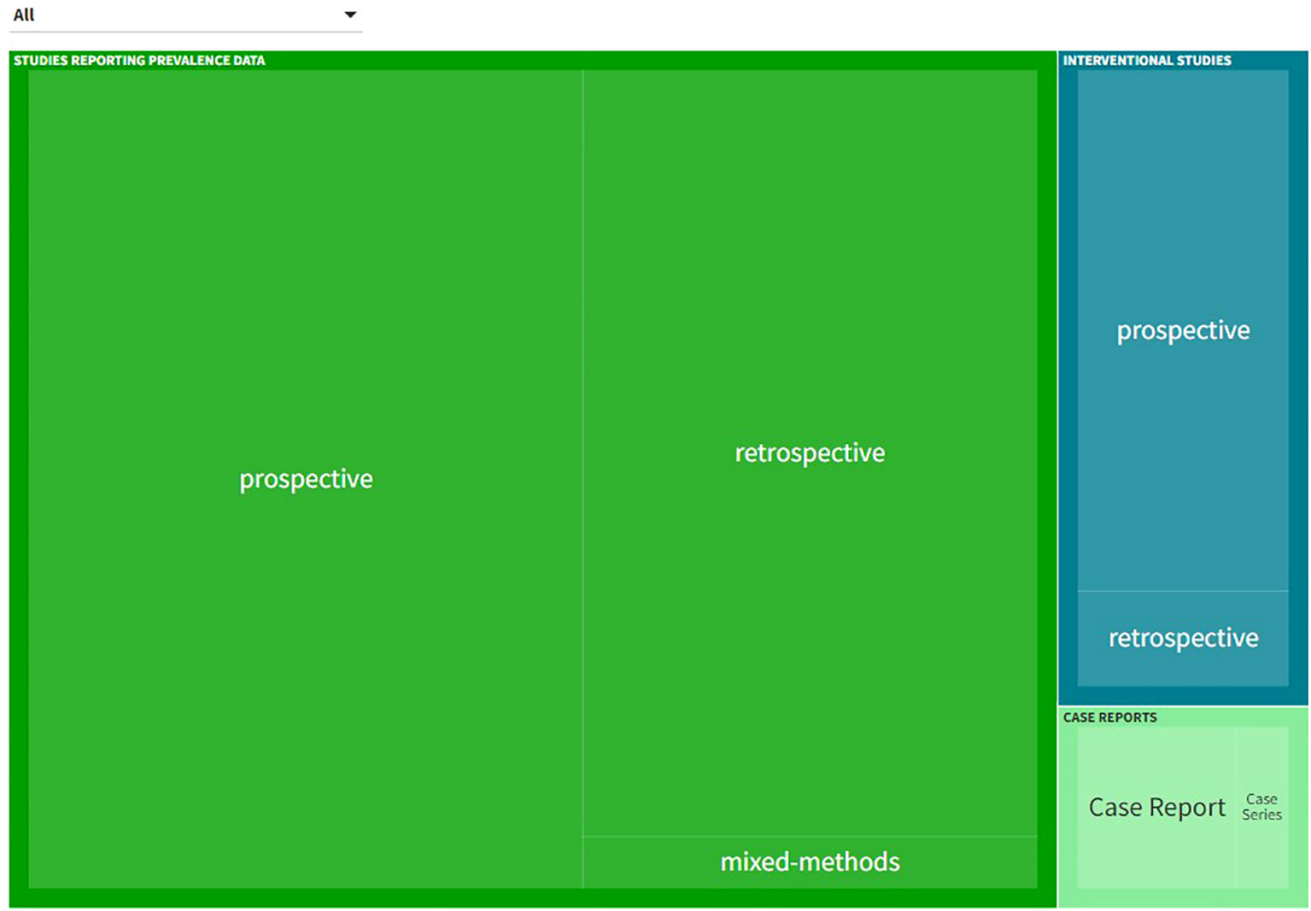
Types of studies and investigated drug-related problem (DRP) types included in the systematic review. An interactive version of this tree map is available online: https://public.flourish.studio/visualisation/17502062/). Users can further explore the investigated DRP types using the interactive tree map (**Figure 4**). Created with flourish.studio (https://flourish.studio).

**Figure 4:**
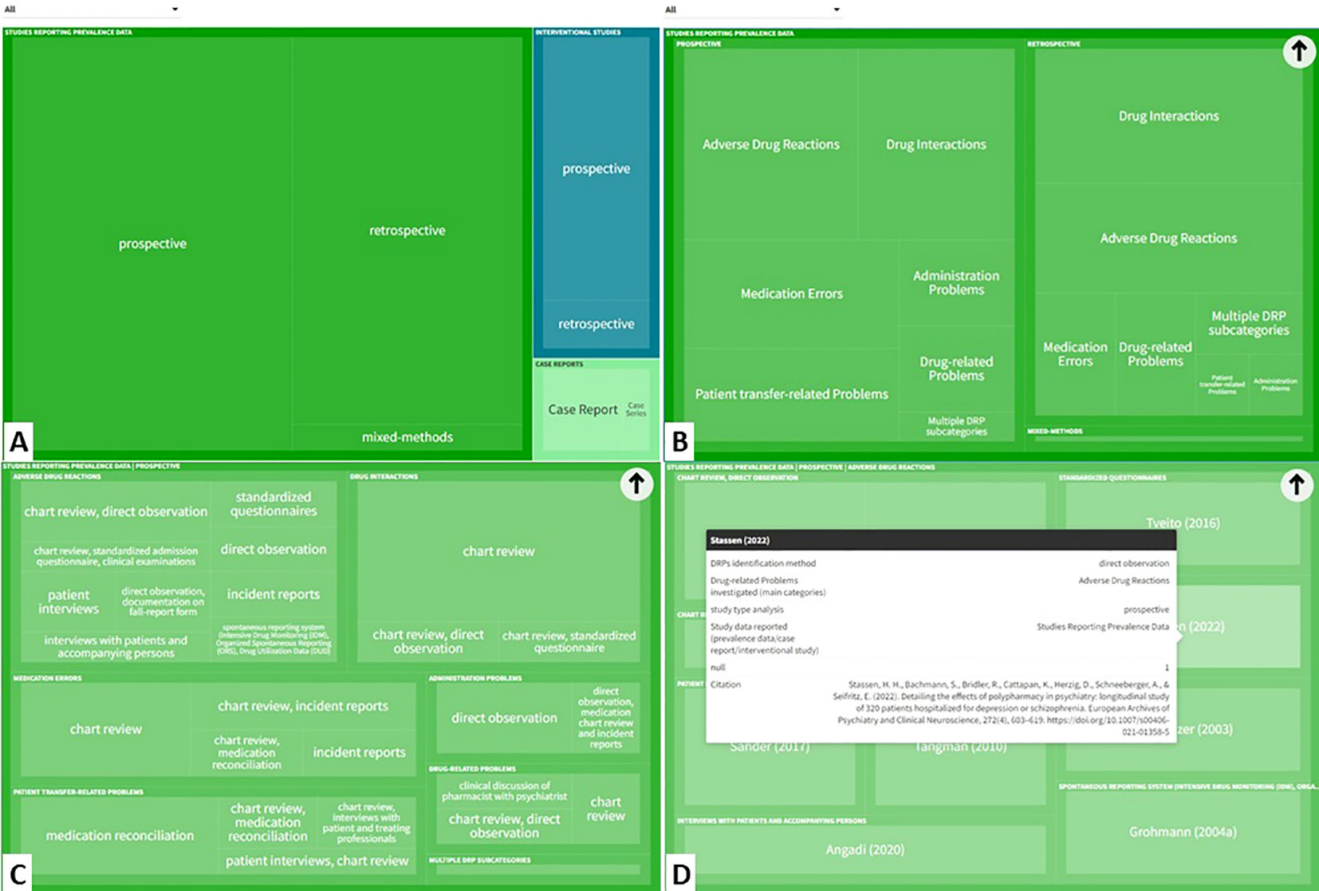
The interactive tree map available online can be explored by users in a hierarchical order: **(A)** Study type > **(B)** DRP categories investigated > **(C)** DRP detection method > **(D)** Individual references reporting this category/subcategory/variable permutation). (https://public.flourish.studio/visualisation/17502062/). Created with flourish.studio (https://flourish.studio). DRP, Drug-related problem.

The characteristics of studies reporting prevalence data, interventional studies and case reports are presented in **Figure 3**. An interactive online version of this tree map is also available (https://public.flourish.studio/visualisation/17502062/). Users can further explore the investigated DRP types and DRP detection methods using the interactive tree map (**Figure 4**).

### 3.2 Quality and bias assessments

#### 3.2.1 DRP reporting quality assessment

The quality assessment of all included studies except the four case reports based on the criteria established by Allan and Barker (48) resulted in a median score of 8 points (out of 12 possible points; interquartile range, IQR: 2, range: 2-11). 82 out of 84 reports (97.6%) clearly describe the aims and objectives of the study. 78 of the reports (92.9%) describe the setting and 81 (96.4%) the data collection method. Regarding the reporting quality of DRPs, fewer studies fulfilled the criteria: 60 reports (71.4%) included a definition for their assessed DRPs, 18 reports (21.4%) specified DRP categories and used a validated classification system whereas 40 (47.6%) specified DRP categories but did not use or name a validated classification system. 35 reports (41.7%) defined the respective DRP categories. For 17 reports (20.2%), DRP categories and their definitions were no applicable criteria as no different DRP categories were assessed in these studies. A clearly defined denominator (e.g. percentage rate of DRPs as number of actual DRPs divided by number of prescriptions/patients or rate of DRPs per 1000 patient days) was present in 50 studies (59.5%). For one study, it was not an applicable criterion as the study assessed an intervention for the improvement of medication adherence without reporting any prevalence data (129). 21 studies (25%) used a validity or causality scale and 42 (50%) reported DRP observation by a pharmacist or senior clinical pharmacologist as validity measures to confirm the occurrence of DRPs. Thus, a total of 63 of the 84 reports (75%) included in the review, applied a validity measure. Reliability measures (e.g. inter-rater reliability assessment) were used in 30 articles (35.7%). Finally, in 68 of 84 reports (81%) study limitations were considered.

For all 31 articles which reported the prevalence of DRPs and medication errors including prescription errors in general (as opposed to only certain DRP subtypes), detailed results of the quality assessment are presented in **Table 4**. For these articles, a median score of 8 points (out of 12 possible points; IQR: 3, range: 4-11) was achieved.

**Table 4:** Quality assessment of included studies which reported the prevalence of drug-related problems (DRPs) and medication errors including prescription errors, based on the criteria established by Allan and Barker (48).

Criteria	Aims	Setting	Data collection method	DRP definition	DRP categories specified 1* 2*	DRP categories defined	Deno-minator	Validity measure 1^a^ 2^b^	Reliability measure	Limi-tations	Score
Ilickovic et al. (130)	1	1	1	0	2	0	0	1	1	1	8
Leherle et al. (55)	1	1	1	1	1	1	0	1	0	0	7
Wolf et al. (123)	1	1	1	1	2	0	1	1	1	1	10
Higuchi et al. (57)	1	1	1	0	0	0	1	0	0	1	5
Soerensen et al. (131)	1	1	1	1	1	1	0	1	1	1	9
Noblot-Rossignol et al. (60)	1	1	1	0	0	0	1	1	0	0	5
Kuzzay et al. (114)	1	1	1	1	1	1	0	1	0	1	8
Kilimann (63)	1	1	1	1	1	1	0	1	0	1	8
Lizer et al. (65)	1	1	1	1	1	1	1	1	1	1	10
Oliveira et al. (68)	1	1	1	1	1	0	0	1	0	1	7
Keers et al. (69)	1	1	1	1	1	1	1	1	1	1	10
Prins et al. (70)	1	1	1	1	1	1	1	1	1	1	10
Heck et al. (96)	1	1	1	1	1	1	1	1	1	1	10
Jayakumar et al. (72)	1	1	1	1	2	0	1	1	1	1	10
Keers et al. (75)	1	1	1	1	2	1	1	1	1	1	11
Jayaram et al. (78)	1	1	1	1	2	0	1	1	1	1	10
Bord et al. (79)	1	1	1	1	1	0	1	1	1	0	8
Rothschild et al. (13)	1	1	1	1	1	1	1	0	1	1	9
Ito et al. (102)	1	1	1	1	0	0	1	0	0	1	6
Kibsdal et al. (103)	1	1	1	1	1	0	1	1	0	1	8
Soerensen et al. (4)	1	1	1	1	1	1	1	0	1	1	9
Grasso et al. (118)	1	1	1	1	1	0	1	1	1	1	9
Grasso et al. (135)	1	0	1	1	1	0	1	1	0	1	7
Schröder et al. (105)	1	1	1	0	1	0	0	1	0	1	6
Brownlie et al. (84)	1	1	1	1	0	0	0	0	0	1	5
Nelson et al. (106)	1	1	1	1	1	0	1	0	0	1	7
Soerensen et al. (86)	1	1	1	1	2	1	1	1	0	1	10
Vermeulen et al. (107)	1	1	1	1	1	1	1	0	1	1	9
Alshahrani et al. (87)	1	1	1	0	0	0	0	1	0	0	4
Singh et al. (88)	1	1	1	1	2	1	0	1	0	0	8
Pratheeksha et al. (110)	1	1	1	1	0	0	0	0	0	0	4

1*, no validated classification system used or named; 2*, validated classification system used; 1^a^, observation by a pharmacist or senior clinical pharmacology physician; 2^b^, validity/causality scale used (e.g. for ADR Naranjo algorithm, UKU scale).

#### 3.2.2 Study type specific quality assessment

In the study type specific quality assessments using the JBI critical appraisal checklists for studies reporting prevalence data, cohort studies and case reports a low risk of bias with a score of more than 70% was achieved for all study types.

In 70 studies reporting prevalence data of DRPs; a median score of 77.8% with 7 out of a maximum of 9 points (IQR: 2, range: 2-9) was reached.

A median score of 72.7% with 8 out of a maximum of 11 points (IQR: 2,75, range: 3-10) was achieved in 14 articles reporting the results of cohort studies.

The four case reports achieved a median score of 100% with 8 out of 8 points in the risk of bias assessment (IQR: 0.25, range: 7-8). Only one report of a case series of adverse effects requiring discontinuation of one or more of the medications in combination therapies with monoamine oxidase inhibitors and other antidepressants or stimulants (119) did not clearly describe the patients’ history and presented it as a timeline. All other criteria were met in all four articles.

#### 3.2.3 Risk of bias assessment

The search of grey literature was not comprehensive. Therefore, it is possible that other studies assessing the prevalence of different types of DRPs and interventions to solve them have been conducted but were not identified in this review. Furthermore, it is possible that interventions without a positive effect on reducing the prevalence of DRPs were not published by the authors or published in journals which were not indexed in the databases searched for this review, and were therefore omitted from this review.

This systematic review was assessed for bias regarding the included interventional studies by the AMSTAR 2 critical appraisal tool (44). The completed form is available online in the **Supplementary Material**.

#### 3.2.4 Certainty assessment

The final gradings of available evidence are included in the summary of findings table (**Table 5**). Most studies were observational studies reporting prevalence data of specific subtypes of DRPs. Only one study was designed as a randomized, controlled trial (127). Furthermore, different methods to detect DRPs were used (incident reports, chart review, direct observation, patient interviews) by data collectors with different professional backgrounds (e.g. clinical pharmacists, clinical pharmacologists, psychiatrists, nurses). Therefore, the identified prevalence rates of DRPs varied widely among studies. According to GRADE, the certainty of available evidence was rated as very low among all DRP subtypes.

**Table 5:** Summary of findings table of drug-related problems (DRPs) in inpatient psychiatry based on the GRADE system (52).

Category	Results from included studies	Grading of evidence
Overall prevalence of DRPs/MEs/PEs		
DRPs	32-948 DRPs per 100 patients/medication reviews (72, 78, 87, 88, 96, 103, 105, 110, 123) 63-87.1 per 1000 patient-days (123)	Very low
MEs	0.3-7032 per 100 patients (4, 13, 78, 93, 107, 118) 0.8-1,515.2 per 1000 patient-days (13, 57, 78, 93, 102, 118) 0.4-174.7 per 1000 opportunities for error (4, 78, 104)	Very low
PEs	15-2636 per 100 patients (4, 79, 86, 114, 118, 131) 37.5-300 per 1000 opportunities for error (4, 69, 75, 79, 131) 0.06-165 per 1000 patient-days (13, 57, 118)	Very low
Prevalence of DRP categories		
Adverse drug reactions	1.3-213 per 100 patients (7, 13, 21, 61, 64, 66, 67, 71–74, 77, 87, 88, 93, 95, 96, 98, 99, 103–105, 107, 109, 110, 113, 123, 130) 0.3-19.2 per 1000 patient-days (13, 93, 100, 104, 123)	Very low
Drug interactions	2.2-1403 per 100 patients (13, 54, 56, 59, 63, 72, 76, 77, 81–83, 86, 88, 90, 91, 94, 96, 97, 101, 103, 105, 108, 110–112, 116, 117, 123, 128, 130, 131, 134) 1.8-3.6 per 1000 patient-days (13, 123) 88 (131) -1500 (63) per 1000 opportunities for error	Very low
Drug selection inadequate	10-123 per 100 patients (86, 87, 96, 103, 105, 123, 130) 15.7 per 1000 patient-days (123)	Very low
Dose selection inadequate	1-88 per 100 patients (13, 72, 96, 103, 105, 130)	Very low
Time of intake inadequate	1-14.6 per 100 patients (103, 123) 3.5 per 1000 patient-days (123)	Very low
Treatment duration	0.5-28.6 per 100 patients (103, 123, 130) 1.3% of prescriptions per patient (79) 1.9-4.2 per 1000 patient-days (123)	Very low
Dosage form inadequate	0.5-11.8 per 100 patients (72, 130) 0.3 per 1000 patient-days (130)	Very low
Patient-related drug use problems	14.3-28.8 per 100 patients (90, 130)	Very low
Storage inadequate	Not studied	No evidence
Non-adherence/Non-compliance	12.8-57.5 per 100 patients (65, 123, 127, 133) 3.4 per 1000 patient-days (123)	Very low
Dispensing	0.04-10 per 1000 patient-days (57, 102, 118) 27.8-134.3 per 1000 opportunities for error (4)	Very low
Administration (Drug use)	0.14 to 1439 per 100 patients (13, 53, 80, 85, 93, 96, 118, 132) 0.8-997 per 1000 patient-days (13, 57, 93, 102, 118) 33-418 per 1000 opportunities for error (4, 80, 85, 124)	Very low
Patient transfer related (Medication reconciliation)	2.6-501 per 100 patients (4, 13, 55, 60, 65, 68, 70, 84, 103, 105, 106, 118, 123, 135) 2.1-344 per 1000 patient-days (13, 118, 123) 226-254 per 1000 opportunities for error (4, 68)	Very low
Other causes	*No or inadequate TDM:* 6.7-82.1 per 100 patients (87, 105, 123) 1.9-6.0 per 1000 patient-days (123) *Complex therapy regimen:* 29.1 per 100 patients (123) 7.7 per 1000 patient-days (123)	Very low
Problems with self-medication	Not studied	No evidence

MEs, Medication errors; PEs, Prescribing errors.

The included reports of interventional studies did not present comparable clinical effect sizes and the tested interventions were not directly comparable by the reported outcome measures. Therefore, the interventions were not included in the summary of findings table.

### 3.3 Prevalence of drug-related problems in inpatient psychiatry

Summaries of studies reporting the prevalence of DRPs or MEs in general are presented in **Table 6**. Their overall prevalence is presented in the summary of findings table (**Table 5**).

**Table 6:** Summary of studies reporting the overall prevalence of drug-related problems (DRPs) and medication errors (MEs) in inpatient psychiatry.

Study	Study design	Number of participants (female, %)	DRP identification method	Prevalence of DRPs/MEs	Prevalence of manifest DRPs/MEs per patient (of all DRPs/MEs)	Preventability	Quality assessment after Allan and Barker (48), max: 12 points
Interventional studies							
Ilickovic et al. (130)	prospective, interventional ‘before-and-after’ study	99: I: 49 (24.5%) C: 50 (24%)	medication review from patient records by CP	1.4 ± 0.7 DRPs per patient	73.5% of patients had at least one manifest DRP, 56.3% of DRPs were ADRs	Not assessed	8
Wolf et al. (123)	prospective, non-randomized, open, controlled trial	265: I: 131 (46.6%) C: 134 (32.1%)	medication reconciliation by two CPs at admission, weekly medication reviews during hospitalization, at discharge, and 3 months after	DRPs at admission: I: 63.0 per 1000 patient-days, 3.0 ± 2.7 per patient C: 87.1 per 1000 patient-days, 3.1± 2.6 per patient Unresolved DRPs at follow-up: I: 5.8 per 1000 patient days, 0.4 ± 0.9 per patient C: 76.9 per 1000 patient days, 2.3 ± 2.1 per patient	I/C: 41.7%/39.9% of all DRPs relevance of DRPs: I/C: minor: 43.8%/43.8% moderate: 46.9%/46.9% major: 7.6%/10.7%	potential + preventable ADEs (unsolved): I/C: 58.3% + 3.0% (12.1% + 16.7%)/ 60.1% + 3.6% (72.2% + 80%) non-preventable ADEs (unsolved): I/C: 13.6% (5.6%)/10.7% (35.6%)	10
Non-interventional studies							
Heck et al. (96)	retrospective cohort study	230 (63.0%)	chart review by physicians	9.5 ± 8.2 DRPs per patient, 94.3% of patients with ≥1 DRP	Not assessed	Not assessed	10
Jayakumar et al. (72)	prospective observational study	198 (26.8%)	chart review and direct observation by academic pharmacist	1.0 DRP per patient, 51.5% of patients with ≥ 1 DRP	19.7% of patients had ≥1 ADR (30.2% of all DRPs were ADRs)	Not assessed	10
Pratheeksha et al. (110)	retrospective observational study	180 (29.4%)	chart review	1.3 DRPs per patient	0.35 ADRs per patient (26.7% of all DRPs were ADRs)	Not assessed	4
Kibsdal et al. (103)	retrospective study	526 medication reviews (not reported)	chart review by CPs	2.3 DRPs per medication review including those without DRPs, 2.5 ± 2.8 DRPs per review with ≥1 DRP, 86.6% of reviews with ≥1 DRP	0.29 side effects per medication review	Not assessed	8
Alshahrani et al. (87)	prospective study	420 (not reported)	chart review by CPs	2.0 DRPs per patient	0.05 ADRs per patient	Not assessed	4
Singh et al. (88)	prospective, observational study	286 (38.8%)	clinical discussion of pharmacist with psychiatrist	0.3 DRPs per patient	0.09 ADRs per patient (29.1% of all DRPs were ADRs)	ADRs caused by DDIs: probable causality: 50% suspected causality: 50%	8

I, intervention group; C, control group; CP, clinical pharmacist; ADE, Adverse drug event; ADR, Adverse drug reaction; DDI, Drug-drug interaction.

**Figure 5:**
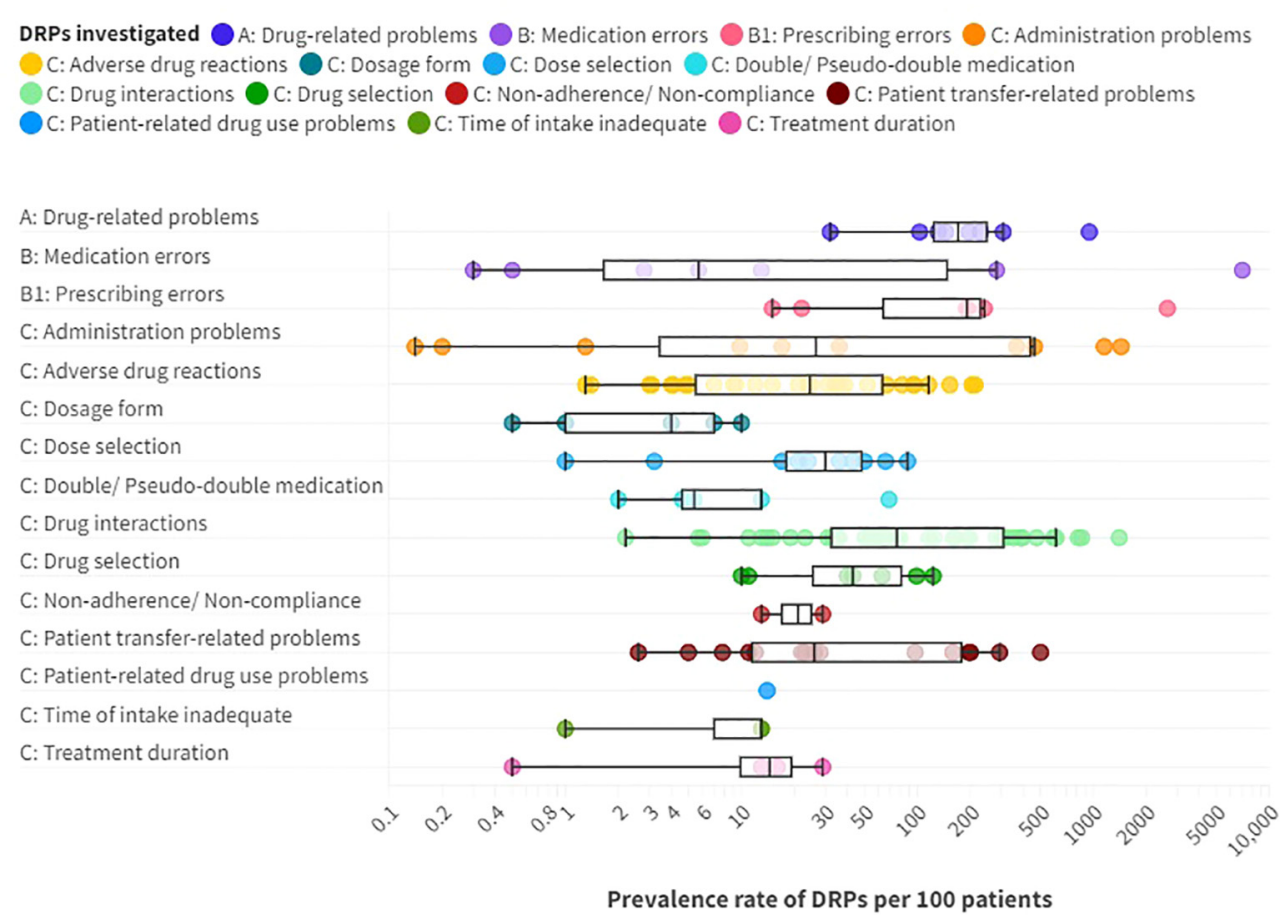
Prevalence rates per 100 patients of A: drug-related problems (DPRs), B: medication errors such as B1: prescribing errors, and C: DRP subtypes reported in the included articles providing prevalence data. Box plot with a logarithmic scale including first quartile, median and third quartile for each DRP category. An interactive online version is also available: https://public.flourish.studio/visualisation/17490375/. Created with flourish.studio (https://flourish.studio).

The prevalence rates of DRPs, MEs and DRP subtypes per 100 patients reported in the included studies are presented as a box plot including first quartile, median and third quartile in **Figure 5**. Users can further explore details of the investigated DRPs, DRP detection methods and the study specific data collectors in an interactive version of this chart, available online (https://public.flourish.studio/visualisation/17490375/).

Overall, DRPs were reported with a prevalence range of 32 to 948 per 100 patients or medication reviews (72, 78, 87, 88, 96, 103, 105, 110, 123) and 63 to 87 per 1000 patient-days (123).

The highest prevalence rates of MEs were reported by Bord et al. (79) with 2636 PEs and Grasso et al. (118) with 7032 MEs per 100 patients, respectively. Furthermore, a high prevalence of 948 DRPs per 100 patients was reported by Heck et al. (96). As MEs, including PEs, and DRPs comprise different subtypes of DRPs, it is evident that the reported prevalence rates were higher than for specific DRP subtypes. The median prevalence rate per 100 patients, calculated from the included studies without weighing in different sample sizes and methodological differences, was highest for PEs (191 per 100 patients) with a range of 15-2636 per 100 patients (4, 79, 86, 114, 118, 131), followed by DRPs (170.5 per 100 patients) (72, 87, 88, 96, 103, 110, 123, 130) with a range of 32-948 DRPs per 100 patients or medication reviews (72, 78, 87, 88, 96, 103, 105, 110, 123).

PEs were identified in 3.8% (69) to 30% (79) of prescriptions (37.5-300 per 1000 opportunities for error) and on 0.06 (57) to 165 (118) of 1000 patient-days (4, 13, 57, 69, 75, 79, 118, 131). Missing dosage forms were identified as a subtype of PEs with a wide range of 0.8% of newly written or omitted items (69) to 89.4% of prescriptions (63) and on 56 of 1000 patient-days (118). While some hospitals used paper charts for drug prescriptions, others have implemented computerized physician order entry (CPOE) systems with or without integrated clinical decision support systems (CDSS) which allow physicians to prescribe on digital medication charts (136). CDSS generate automatic alerts to warn prescribers of possible hazards resulting from the prescribed medication, such as DDIs (136). CPOE-operating errors were identified as the cause of PEs in 7.4% of patients (114). The highest prevalence rate of inappropriate prescriptions, which were considered as PEs for simplification, were reported by Bord et al. (79). They included both formal and content-related problems such as DDIs and dosage problems. However, no prevalence rates per 100 patients could be calculated for the PE subcategories using the reported data. Therefore, only the overall prevalence of 2636 inappropriate prescriptions per 100 patients was included in the data synthesis in **Figure 5**.

Of note, the median prevalence rate of MEs (5.7 per 100 patients) (4, 13, 78, 107, 118) was lower than of multiple DRP subtypes, which can be explained by the wide range of ME prevalence rates reported in the included studies of 0.3 and 0.5 identified through incident reports (78) to 7032 per 100 patients identified by chart review and prospective self-reports of dispensing errors (118) or 0.8 to 1515 per 1000 patient-days (13, 57, 78, 93, 102, 118) or 0.4 to 174.7 per 1000 opportunities for error (4, 78, 104). The highest prevalence rates were identified through direct observation (4).

Regarding clinical implications of MEs, Rothschild et al. (13) reported a serious ME rate of 6.3 per 1000 patient-days and 7.7 per 100 patients.

#### 3.3.1 What are the most frequent DRPs and DRP subtypes in adult psychiatric inpatients?

Among the DRP subtypes, potential clinically relevant DDIs were reported with the highest median prevalence rate of 76.5 per 100 patients (13, 54, 56, 59, 63, 72, 76, 77, 81–83, 86, 88, 90, 91, 94, 96, 97, 101, 103, 105, 108, 110–112, 116, 117, 123, 128, 130, 131, 134) with a wide range of 2.2 (13) to 1403 per 100 patients (63), 1.8 to 3.6 per 1000 patient-days (13, 123) and 88 (131) to 1500 (63) per 1000 prescriptions.

Jabeen et al. (82) reported the prevalence of different drug interactions, including DDIs (72 per 100 patients), drug-food interactions (68 per 100 patients), drug-alcohol interactions (136 per 100 patients), and drug-tobacco interactions (28 per 100 patients) with an overall prevalence rate of 304 per 100 patients (82).

Other studies focused on potentially clinically relevant pharmacokinetic DDIs (pkDDIs) involving CYP-450-metabolism in regular prescriptions (CYP3A4: 27.2-36.1% of patients (54, 83), CYP2D6: 9.3-34.7% of patients (54, 83), CYP2C19: 6.5% of patients (54), CYP1A2: 3.2% of patients (54), CYP2C9: 2.3% of patients (54)) or in drugs prescribed to be taken as needed (CYP2D6: 25% of patients (101), CYP3A4: 11% of patients (101)).

DDIs of drugs with the potential to lead to prolongation of the patients’ QT-interval (QT-DDIs) were assessed in three studies with reported prevalence rates of 19.1 to 116 per 100 patients (59, 97, 117). Javelot et al. (76) analyzed hazardous or contraindicated DDIs in elderly and non-elderly psychiatric inpatients and reported that involvement of polypharmacy with a QT-prolonging antipsychotic was significantly higher in non-elderly patients, accounting for 65.4% (9.5 per 100 patients) of hazardous DDIs in non-elderly patients and 23% (3.9 per 100 patients) of hazardous DDIs in elderly patients (p=0.002) (76). ADRs caused by QT-DDIs are reported below in the paragraph on ADR prevalence rates.

Furthermore, DDIs with antidepressants were identified in 27% of patients (111), drug-disease interactions were found in 15 to 36.1% of patients (86, 96, 131) and 3.2% of prescriptions (131), and drug-genotype interactions were reported for 20% of patients (56).

The second highest median prevalence rate with 43 per 100 patients (86, 87, 96, 103, 105, 123, 130) with a range of 10 (130) to 123 per 100 patients (96) concerned drug selection, including e.g. omission of potentially useful drugs (1.1 to 22.6 per 100 patients, 0.1-6.0 per 1000 patient-days (123)), disregard of drug allergies (3 per 100 medication reviews (103)), missing indications (10.2 to 50 per 100 patients (79, 96, 123), 0.7-4.5 per 1000 patient-days (123), 45 per 1000 prescriptions (131)), disregard of contraindications (0.5 to 7.8 per 100 patients (79, 96)), and inappropriate choice of drugs according to guidelines (2.2 to 46.9 per 100 patients (63, 103)).

The third highest median prevalence rate with 30 per 100 patients (13, 72, 86, 87, 96, 103, 105, 123, 130, 131) with a range of 1 (72) to 88 per 100 patients (130) was reported for dose selection problems. On one hand, dosages were too low with a prevalence rate of 4 to 26.5 per 100 patients (105, 123), 1.6% of prescriptions per patient (79) or 1.7 to 5.9 per 1000 patient-days (123). On the other hand, dosages were too high in 6.4 to 27% of patients (86, 105, 123, 131), 3.7% of prescriptions (131), 14.7% of prescriptions per patient (79) or 1.5-1.7 per 1000 patient-days (123). Studies correlating TDM results with specific DRP subtypes are summarized separately below.

Dose selection was inadequate due to renal or hepatic insufficiency in 31.3% of all patients and in 10.2% of prescriptions on a gerontopsychiatric ward (63). Furthermore, inadequate dosing frequencies were present in 4.4 to 25 of 100 patients (123), 2.9% of prescriptions per patient (79) or 6.7 times per 1000 patient-days (123).

Similarly, a median prevalence rate of 26.5 per 100 patients (13, 53, 80, 85, 93, 96, 118, 132) and a range of 0.1 (132) to 1439 (118) was identified for administration problems (0.8-997 per 1000 patient-days (13, 57, 93, 102, 118), 33-418 per 1000 opportunities for error (4, 80, 85, 124)). Among the reported subtypes of administration errors, 0.8 to 10.2% of patients (85, 93) got or took drugs at the wrong times on 0.2 to 8.4 of 1000 patient-days (57, 93, 102) or in 2.9 per 1000 opportunities for error (85). Drugs were under-used or under-administered in 1.8 to 10.3% of patients (13, 80, 85, 93) on 0.1 to 13.4 of 1000 patient-days (13, 57, 93, 102) or in 12.5 per 1000 opportunities for error (85). The wrong dose was administered to 1.4 to 1.8% of patients (85, 93) on 0.1 to 1.8 of 1000 patient-days (93, 102) and in 6.0 per 1000 opportunities for error (85).

The wrong drug was administered to at least 0.7 to 0.8% of patients (85, 93) on 0.1 to 0.9 of 1000 patient-days (57, 93, 102) and in 2.6 per 1000 opportunities for error (85), as identified by incident reports. Lastly, drugs were identified as administered to the wrong patient in 0.06% (through direct observation) (85) to 0.17% (through incident reports) of patients (85, 132) on 0.1 out of 1000 patient-days through incident reports (57) and in 0.2 per 1000 opportunities for error through direct observation (85).

Stubbs et al. (53) and Haw et al. (80) studied unauthorized dose form modifications (crushed/opened oral solid doses) and identified problems in 7.8 to 11.2% of solid oral drugs (53, 80), in 44.0% of crushed/opened solid doses (53) and 3.7 times per patient (53). Oral solid drugs were crushed or opened contrary to manufacturer’s advice in 4.5% (53).

Dispensing problems were identified with a prevalence of 0.04 to 10 per 1000 patient-days (57, 102, 118) and, depending on the detection method, in 27.8 per 1000 opportunities for error through direct observation of nurses and in 134.3 per 1000 opportunities for error through control of drugs dispensed by nurses (4).

A median prevalence rate of 26 per 100 patients (4, 13, 55, 60, 65, 68, 70, 84, 103, 105, 106, 118, 123, 135) was identified for patient transfer-related problems, including medication discrepancies at admission and discharge, with a range of 2.6 (13) to 501 per 100 patients (118). In the included studies, professionals with different backgrounds used different methods for DRP identification and consequently identified different prevalence rates of medication discrepancies. While physicians identified medication discrepancies in 2.2 to 4.7% of admissions through chart review (13, 105), they found discrepancies in 78% of patients using a structured medication history including a patient interview, brown bag review and medication reconciliation (70). Medication discrepancies at admission were identified in 12 to 53% of patients through medication reconciliation by pharmacists (55, 60, 103, 123), and in 56.2% through medication reconciliation by pharmacy technicians (84). Lizer et al. (65) reported that clinical pharmacists identified significantly more medications on admission than nurses (p<0.05). 17% of discrepancies at admission had clinical consequences (ADEs) in 24% of all patients (70).

At discharge, medication discrepancies were identified in 23% of patients on handwritten paper charts by a board certified psychiatric pharmacist and pharmacy students on senior rotations (106). Similarly, 22% of handwritten discharge lists contained errors identified by pharmacists compared to 8% of discharge lists generated with personal digital assistants (135). Nurses identified medications discrepancies in 28% of discharge summaries (4).

ADRs were reported with a median prevalence of 24.5 (7, 13, 21, 61, 64, 66, 67, 71–74, 77, 87, 88, 93, 95, 96, 98, 99, 103–105, 107, 109, 110, 113, 123, 130) and a range of 1.3 (95) to 213 per 100 patients (98) on 0.3 to 19 of 1000 patient-days (13, 93, 100, 104, 123). Sander et al. (61) identified more ADRs on a gerontopsychiatric ward (1.8 per patient) than in clinical social psychiatry (0.8 per patient). Rothschild et al. (13) identified 10 manifest ADEs per 1000 patient-days. Alshehri et al. (21) identified ADEs with confirmed definite or probable causality in 20.7% of patients and on 4.6 per 1000 patient-days. Drugs were discontinued due to ADEs in 8.6 to 16.1 of 100 patients (66, 123) and on 3.7 per 1000 patient-days (123). Severe ADRs were identified in 0.06 to 9.3 per 100 patients (66, 67, 77, 93, 95, 99).

ADRs were caused by DDIs in 1.1 to 12.5 per 100 patients (88, 109, 110, 134). 6.6% of patients with QT-DDIs developed QT-prolongation of ≥450 [men] or 470 [women] ms or an increase of ≥ 30 ms within 14 days after starting a new QT-prolonging drug (117). Rodríguez-Leal et al. (7) did not report a prevalence rate of QT-DDIs per 100 patients, they did however find dangerously prolonged QTc-intervals in 4% of psychiatric inpatients.

ADRs were caused by one or more PIM-prescriptions in 56.3 per 100 geriatric psychiatric inpatients (71). 23% of geriatric psychiatric inpatients experienced severe ADRs caused by PIM (71).

Akpinar et al. found an incidence for leukopenia and agranulocytosis of 5.4% of patients who used clozapine with another antipsychotic, in 1.1% of patients (n=1) these ADRs were fatal (92). ADRs following high-dose olanzapine treatment (> 40 mg) were identified with a prevalence rate of 95.6 per 100 patients, 53.8% of patients had at least one ADR, and 5.7% were severe (death, neuroleptic malignant syndrome, serious extrapyramidal symptoms) (113). One patient died after taking an overdose of olanzapine, clonazepam and methadone. However, blood concentrations were only reported as high without presentation of specific concentrations (113). As a further type of ADEs, 83 per 100 patients with dementia experienced drug-related falls on a psychogeriatric ward (64).

Only two studies reported prevalence data for non-adherence or non-compliance (90, 123). Wolf et al. (123) identified 13 per 100 patients through medication reconciliation, chart review and patient interviews who did not use prescribed drugs while Buenger et al. (90) supposed that 29 per 100 patients took their drugs irregularly according to TDM measurements.

Problems with drug storage of self-medication were not studied or reported in any of the included articles in psychiatric inpatient settings.

The reported prevalence rates per 100 patients for the other DRP subtypes (inadequate time of intake, patient-related dug use problems, problems with treatment duration or dosage form and (pseudo-) double medication are presented in **Figure 5** and can be explored in the interactive version available online (https://public.flourish.studio/visualisation/17490375/).

#### 3.3.2 Prevalence of DRPs per 1000 patient-days or per 100 opportunities for error

21 studies did not report data from which DRP prevalence rates per 100 patients were computable (20, 57, 58, 62, 69, 75, 89, 92, 100, 102, 115, 119–122, 124–127, 129, 133). However, the included non-interventional studies reported data on prevalence rates per 1000 patient-days or per 100 opportunities for error.

Two Japanese studies reported the prevalence of MEs per 1000 patient-days identified through incident reports (57, 102). Ito et al. (102) reported 0.79 preventable ADEs (pADEs) per 1000 patient-days which solely occurred in the drug administration stage. 24.9% of pADEs were intercepted before reaching the patients. Higuchi et al. (57) identified a hospital-wide ME rate of 2.14 per 1000 patient-days with 94% MAEs, 2.6% PEs and 2.6% dispensing errors. The ME rate for closed wards was 2.31 compared to 0.93 on open wards (57).

Another two studies specifically reported the prevalence of PEs in mental health hospitals (69, 75). 6.3% of all screened prescription items during hospitalization (75), 5.1% of newly written or omitted items at discharge and 81% of discharged patients were affected by at least one PE (69). Of all PEs, during hospitalization 56.2% were rated as clinically relevant (75) and at discharge, 73% were rated as potentially clinically relevant (69). Keers et al. reported that increasing numbers of prescribed items, with a statistical significance at 11 or more items, and the use of an electronic discharge prescription pro forma were associated with an increased risk of making PEs (69).

MAEs were studied in a prospective cohort study and non-significantly reduced from 8.9% of opportunities for error before to 7.2% after the introduction of an automated dispensing cabinet (124). The rate of errors with a clinical effect on the patient remained at 5.4% of opportunities for error without and with the cabinet (124).

Three of the studies, which did not report data from which a prevalence rate per 100 patients could be calculated, assessed ADRs in psychiatric inpatients (20, 62, 100).

In a retrospective study of ADRs in hospitalized psychiatric patients, Thomas et al. (100) identified 0.28 ADRs per 1000 patient-days. 20.4% of these were rated as preventable ADRs (pADRs) and therefore judged as MEs with 47% of pADRs rated as severe, 42% as significant and 10.5% as mild (100).

Among all patients admitted to a gerontopsychiatric ward in Norway, 34% presented themselves with major side effects and 99% of these patients used psychotropic medications (62).

In a younger psychiatric population in Switzerland, at least mild ADRs were identified in 87.3% of patients with schizophrenia (F2) and in 83.5% of depressed patients (F3) (20). ADRs were rated as severe in 39.4% of F2-patients and 37.1% of F3-patients (20).

Specific DDIs (and subsequent ADRs) were studied in three further studies (58, 92, 115).

In a patient sample of 92 patients, 5.4% of patients receiving a combination of clozapine with another antipsychotic (olanzapine, haloperidol, risperidone) developed leukopenia or agranulocytosis (92). One patient (5% of patients) died under combination of clozapine and risperidone because of an infection associated with agranulocytosis (leukocytes= 900/µL) (92). No cases of leukopenia or agranulocytosis were identified for the combinations of clozapine with amisulpride, quetiapine, paliperidone and aripiprazole (92).

In 152 patients taking at least two different drugs with the potential for QT-prolongation, 10 cases of QT-prolongation or a delta QTc of 30 milliseconds or longer (6.6%) were identified (58).

Physician response to a medication alert system in inpatients with levodopa-treated diseases was studied in different departments of an American hospital including psychiatry for five years (115). 44 alerts were triggered in psychiatric patients by the prescription of a dopamine receptor antagonist for inpatients who were already prescribed carbidopa-levodopa or vice versa in the electronic order entry system with a prevalence of inappropriate prescriptions per levodopa order due to drug-disease interactions of 16.1% (115). There was no significant physician response to the alert with a mutually adjusted OR for the discontinuation of inappropriate prescriptions after an alert of 0.12 (95% CI 0.01, 1.48). Only 5.3% of alerts were accepted by the prescribing physicians (115).

Additionally, four case reports were identified which assessed DDIs and severe ADRs caused by DDIs (119–122).

Non-adherence or non-compliance were assessed in two interventional studies without prevalence data and will therefore only be discussed in the section on interventional studies below (127, 129).

#### 3.3.3 Further relevant information from included studies reporting the prevalence or a case of DRPs

Some of the included articles assessed the preventability of ADEs. Rothschild et al. rated 13% of ADEs as caused by MEs and therefore pADEs, whereas 87% were rated as non-preventable with a total rate of 10 ADEs or 1.3 pADEs per 1000 patient-days (13). Similarly, Alshehri et al. rated 81% as non-preventable ADEs (21). All pADEs (19% of all ADEs) resulted from PEs, with a prevalence rate of 2.2 pADEs per 100 patient admissions or 0.5 pADEs per 1000 patient-days (21).

In addition to the preventability of DRPs, the clinical relevance must be assessed for the interpretation of prevalence rates. As an example, Castilho et al. (116) reported an increase in the percentage of patients with one or more DDIs from admission to median length of stay and last prescription before discharge or death (67%, 74.4% and 80.8%, respectively) in institutionalized elderly patients (≥ 60 years). However, the percentage of contraindicated DDIs decreased from 5.1% at admission to 3.3% at the median length of stay and to 1.6% at the last prescription (116).

Another study assessed DDIs, DGIs through pharmacogenomic testing and ADRs and did not find a correlation between the Antidepressant Side Effect Checklist (ASEC) score reduction and medication changes based on phenotypes (p=0.85) (56). However, for patients who completed the trial the ASEC score improved significantly from 11.5 ( ± 8.1) at baseline to 7.2 ( ± 6.0) at follow-up (p=0.0009), showing a significant effect over time (56). It remains uncertain, whether the ASEC score reduction would have been achieved without pharmacogenomic testing as there was no control arm.

A French retrospective study assessed the impact of integrating pharmaceutical expertise in ADR management and reporting of ADRs to a pharmacovigilance center (89). Of note, 51% of ADRs reported to the clinical pharmacist by physicians or patients were caused by pdDDIs and 38% by pkDDIs and a total of 79% of ADRs were rated as potentially iatrogenic. 96% of ADRs were judged as minor or moderate and 4% as major (89). Clinical improvement was observed by a general practitioner or a psychiatrist in 35% of patients due to pharmaceutical interventions (89).

Some studies only reported the prevalence of very specific DRPs, such as drug-related falls (64). Their results were therefore not directly comparable with other studies included in this review. However, they were included in the chart in **Figure 5** if prevalence rates per 100 patients were reported or could be calculated. The specific DRPs studied can be explored online in the section “DRPs investigated (details)” for each data point (https://public.flourish.studio/visualisation/17490375/).

#### 3.3.4 Is there a correlation between DRPs and blood concentrations of drugs?

Seven studies assessed blood concentrations of drugs in the context of DRPs (62, 89, 90, 100, 113, 121, 133).

One article reported that 20% of patients with major side effects had serum concentrations above the reference range for psychotropic drugs, compared to 13.2% of patients with no or minor side effects (p=0.204) (62).

In a Turkish study on the role of clinical pharmacists in compliance of patients with schizophrenic spectrum disorders, plasma levels of clozapine, valproic acid, and lithium were measured during the first week of hospitalization in order to reveal undesirable clinical manifestations, such as possible DDIs, poor therapeutic response, high-dose drug intake (suicide attempt), and noncompliance (133). While all measured plasma levels of valproic acid and lithium were within the therapeutic reference range, 67% of the measured clozapine concentrations were outside the target range (133). The investigators did not explore any types of DRPs in correlation with these plasma concentrations.

In a retrospective study in a German psychiatric department, 62% of serum concentrations were within the therapeutic reference range, 41% within the dose-related reference range and 30% within the therapeutic and dose-related reference range (90). While most studies reported the prevalence of DDIs based on DDI checks on different databases, Buenger et al. calculated the percentage of DDIs that were present in patients’ TDM samples (15%) equaling 31% of patients. 65% of these interactions (20% of patients, 10% of TDM samples) were related to the use of CYP1A2-substrates and cigarette smoke (90). In 14% of TDM samples (29% of patients), a subtherapeutic plasma level was supposedly explained by an irregular intake of drugs (non-compliance) (90).

In one case report, the pkDDI between carbamazepine and quetiapine was studied by measuring quetiapine plasma concentrations because of an insufficient clinical effect of quetiapine in the patient’s manic episode (121). No serum quetiapine was detected during concurrent use of carbamazepine due to induction of CYP3A4. The lack of therapeutic efficacy correlated with the subtherapeutic plasma levels of quetiapine due to the pkDDI with carbamazepine which lasted for at least three weeks after carbamazepine withdrawal (121).

Three further articles used TDM but did not report specific blood concentrations.

In a study on high-dose olanzapine therapy from Denmark, high blood concentrations were measured in a patient who died after taking an overdose of olanzapine, clonazepam and methadone (113).

In another study, the documentation of a toxic serum concentration was listed as one of the preventability criteria for ADRs, e.g. in three cases of phenytoin toxicity in patients who experienced drug-related falls due to missing TDM (100). Overall, 20% of the identified ADRs were rated as preventable (0.06 per 1000 patient-days) (100). However, the percentage of drug plasma concentrations outside the therapeutic reference range involved in pADRs overall was not specifically reported.

Lastly, 12% of ADRs reported to pharmacists by physicians and patients in a study on integrating pharmaceutical expertise in ADR management were related to blood concentrations of clozapine, aripiprazole, lithium, and risperidone outside the therapeutic reference range (89).

### 3.4 Interventions to solve drug-related problems in inpatient psychiatry

**Figure 6:**
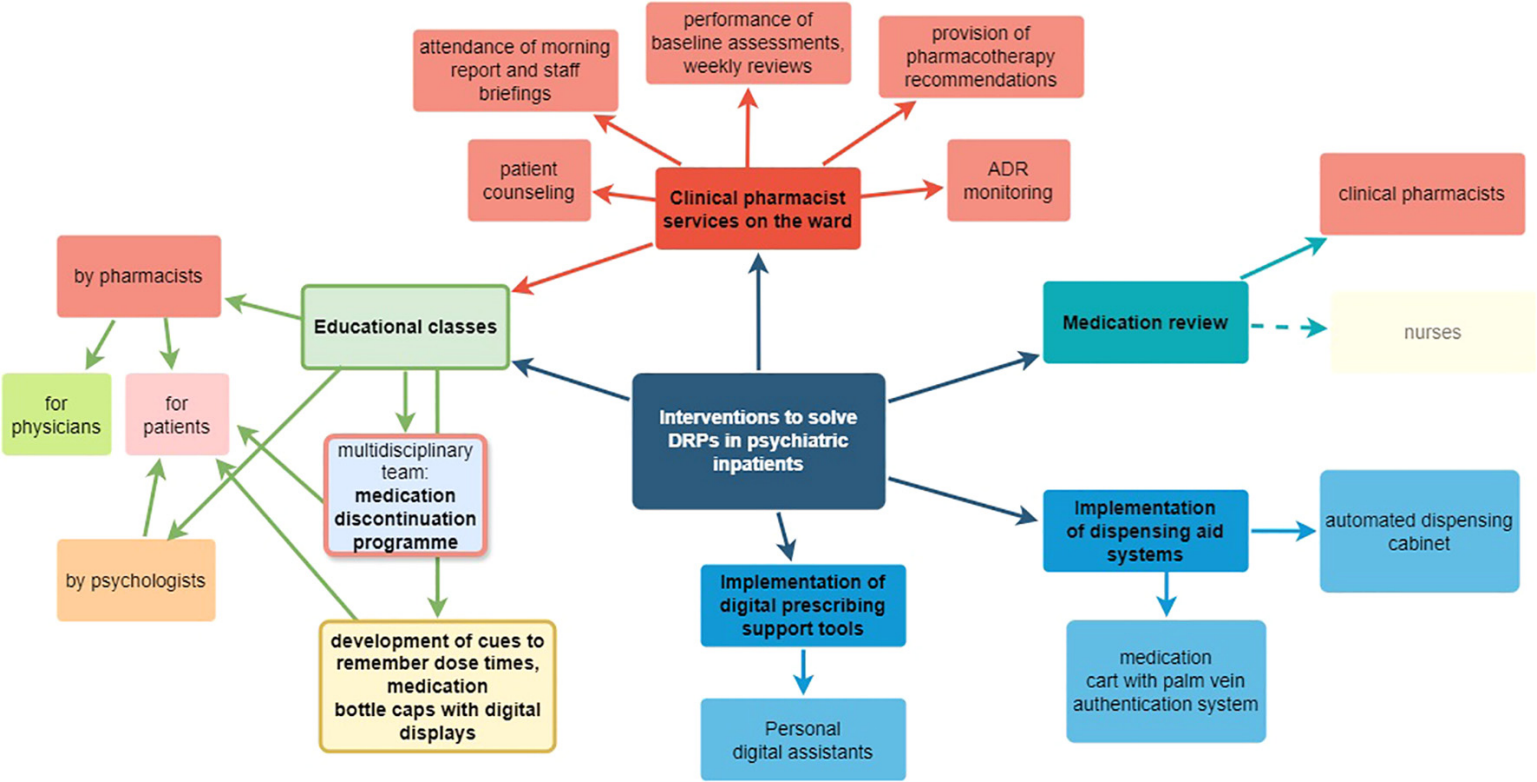
Mind map of clinical intervention types and involved professionals in the included studies. Created with draw.io (https://www.drawio.com/).

The summaries of statistically tested interventions to solve DRPs in inpatient psychiatry are presented in **Table 7**. Since the reported effects were not directly comparable, the interventions and their outcomes are summarized by intervention type in the paragraphs below. An overview of intervention types and professionals involved in the included studies is presented as a mind map in **Figure 6**.

**Table 7:** Summary of studies reporting the results of statistically tested interventions to solve drug-related problems (DRPs) in inpatient psychiatry.

Study	Study design	Number of participants (female, %)	DRPs investi-gated	Description of intervention	Effect of intervention	Unsolved DRPs/MEs after inter-vention	Inter-vention acceptance	Quality assessment after Allan and Barker (48), max: 12 points
Medication review								
Ilickovic et al. (130)	prospective, interventional ‘before-and-after’ study	99: I: 49 (24.5%) C: 50 (24%)	DRPs	medication review from patient records by CP who visited physicians to resolve questions before physician decided whether to accept recommendations	35.2% of DRPs solved, significant reduction in No. of prescribed drugs per patient: T_paired_= -0.263, p=0.002	60.3% with known outcome unsolved: partially solved: 20.6%, not solvable: 39.7%	71.7%	8
Wolf et al. (123)	prospective, non-randomized, open, controlled trial	265: I: 131 (46.6%) C: 134 (32.1%)	DRPs	medication reconciliation by two CPs at admission, weekly medication reviews during hospitalization, at discharge, and 3 months after discharge (follow-up)	significant reduction in No. of unsolved DRPs per patient: -1.8 (95% CI: 1.5–2.1, p<0.001) MAI score significantly reduced compared to C: at discharge: -1.4 points per patient (95% CI: 0.8–2.1, p<0.001), at follow up: -1.2 points (95% CI: 0.6–1.9, p < 0.001)	5.8 DRPs per 1000 patient days, 0.4 ± 0.9 per patient, 12.6% of DRPs	88.6%	10
Soerensen et al. (131)	controlled, interventional before-and-after study	396 (57%): I: 121 (50.8%) C: 275 (n.r.)	PEs (MEs)	medication review by nurses after admission	no significant effect; difference-in-difference of mean number of PEs: -0.23 (95% CI: -1.07 to 0.60), p=0.59 patients prescribed ≥1 PE: OR: 0.61 (95% CI 0.25-1.46), p=0.26, percentage of patients reduced from 65.5% to 53.7%, p=0.14	not reported	34% only 17% of the accepted interventions were PEs identified by senior clinical pharmacology physicians	9
Implementation of digital tools								
Grasso et al. (135)	retrospective study	110 discharge medication lists transcribed by hand, 90 discharge lists generated by personal digital assistants	transfer-related PEs (MEs)	introduction of personal digital assistants for drug prescription including a pharmacology database	significant reduction in No. of discharge medication lists containing errors from 22% to 8% (chi²=4.58, df=1, p<0.05)	8% of medication discharge lists contained errors	not assessed	7
Cottney et al. (124)	prospective before-and-after cohort study	I: 60 medication rounds with 1542 observed opportunities for error C: 60 medication rounds with 1895 observed opportunities for error	MAEs (MEs)	installation of an automated dispensing cabinet on a psychiatric ward	no significant error reduction (p=0.065, 95% CI 0- 3.5%): 1.7% reduction in the MAE rate from 8.9% to 7.2%	7.2% of administered medications erroneous	not applicable	11
Sawa et al. (132)	pre-post observational study	3523	MAEs (MEs)	introduction of a medication cart equipped with a palm vein authentication system	significant reduction in misidentification errors of patients from 0.17% to 0.06% of patients (p<0.0001) MAEs: reduced from 0.20% to 0.14% of patients	1.4 per 1000 patients	not applicable	4
Educational classes								
Hahn et al. (134)	retrospective cohort study	393: I: 200 (52.5%) C: 193 (49.2%)	DDIs, ADRs	educational classes on DDIs by a CP/a physician for internal medicine and pharmacist consultation service on DDIs	significant reduction of potential DDIs per patient (p=0.04): I: 2.2 vs. C: 3.4 reduction of clinically relevant DDIs through CP interventions by 78%; overall reduction of potential DDIs by 44%	44.2% of all DDIs, 22.1% of relevant DDIs	not reported	6
Bertsche et al. (128)	prospective pilot study	30 (66.7%), 10 patients per group (control, intervention PHARMA, intervention PSY)	DDIs, MA	two intervention groups: PHARMA: DDI checks by a pharmacist, clinically relevant DDIs communicated to physician in written form; pharmaceutical counseling (60 minutes/patient) PSY: psychological counseling (60 minutes/patient) on drug treatment	prevalence of DDIs: control/intervention PSY: no change; intervention PHARMA: significant decrease from 2.67 (IQR: 1/4) to 0,63 (0/1,25) DDIs per patient (p<0.05), no significant differences between groups in MARS-score before and after intervention but significant effect over time (p<0.05)	0.63 DDIs per patient	not assessed	6
Cramer et al. (127)	prospective randomized clinical trial	81: I: 41 (15%) C: 40 (12%)	MA	educational sessions on development of cues to remember dose times, medication bottle caps with digital displays showing the number of times the bottle had been opened that day, feedback sessions with patients about adherence pattern	significant improvement of compliance: mean 1-month compliance rates before the first visual calendar feedback session (p=0.023): I: 81 ± 22% C: 68 ± 27% mean overall compliance (p=0.008): I: 76 ± 22%, C: 57 ± 30%	not applicable	not applicable	7
Murasugi et al. (129)	pilot, prospective, interventional cohort study	24: I: 7 (14.3%) C: 17 (11.8%)	MA	medication discontinuation programme (MDP) with psychoeducation in forensic psychiatry by multidisciplinary team	significant increase in DAI-30 (p=0.002): I: before MDP: -2.6 ± 13.2; after MDP: 18.3 ± 9.2, recovery phase: 19.9 ± 8.5 C: no significant difference between initial vs. second evaluation	Not applicable	Not assessed	4
Yalçin et al. (133)	prospective study	40 (37.5%)	MA	patients were followed by CP who analyzed drug efficacy, compliance and ADRs during patient hospitalization and for 4–6 weeks following discharge	significant increase in compliance (MARS) after drug education (p<0.001): 6.60 ± 2.23 to 8.60 ± 1.29, decrease in percentage of patients with poor MA from 30.4% to 17.5%	17.5% of all patients with poor MA	69.6% fewer patients with poor MA	6
Clinical pharmacist services on the ward								
Canales et al. (125)	prospective cohort study	45 (53%)	ADEs	CP services on two adult acute care wards	Adverse-event-rating scales: significant differences in: AIMS (mean ± SD % change in score): 3.5 ± 12.1 (p=0.024), Barnes Rating Scale for Drug-Induced Akathisia: 27.0 ± 52.1 (p=0.042) Simpson-Angus Rating Scale for Drug-Induced Extrapyramidal Symptoms: 21.9 ± 31.9 (p=0.002), BPRS score improvement (percentage of patients, difference between I/C): ≥20%: 93% (p=0.024) ≥30%: 62% (p=0.002) ≥40%: 22% (p<0.001), CGI score improvement (p<0.001)	not assessed	94%	6
Other interventions								
Lima et al. (126)	prospective cohort study	14 (64.3%), slow titration: 7 (57.1%), loading: 7 (71.4%)	ADRs to divalproex sodium	slow titration or loading of divalproex sodium	significantly greater incidence of treatment emergent somnolence in the slow titration group (p=0.0210), no other significant differences	not applicable	not applicable	5

I, Intervention group; C, Control group; ADRs, Adverse drug reactions; AIMS, Abnormal Involuntary Movement Scale; ASEC, Antidepressant Side Effect Checklist; BPRS, Brief Psychiatric Rating Scale; CGI, Clinical Global Impressions Scale; CI, Confidence interval; CP, Clinical pharmacist; DAI-30, Drug Attitude Inventory-30 score; DDI, Drug-drug interaction; DGI, Drug-genotype interaction; IQR, Interquartile range; MA, Medication adherence; MAE, Medication administration error; MAI, Medication Appropriateness Index; MARS, Medication Adherence Rating Scale; No., Number; Not solvable, no need or possibility to solve the problem; OR, Odds ratio; PE, Prescribing error; QT-DDIs, Drug-drug interactions of drugs with the potential to prolong the patient’s QT interval.

Interventions were conducted at least partly by pharmacists in 7 of the 13 identified studies (123, 125, 128, 130, 133, 134). Other interventions were conducted by nurses (131), a multidisciplinary team (129), a psychologist (128) or through implementation of digital tools (124, 132, 135). In one article, the profession of the investigators was not reported (127).

#### 3.4.1 Medication reviews

Six of the included interventional studies identified DRPs or DRP subtypes at least partly by chart and medication review (123, 128, 130, 131, 134, 135). In four articles, interventions were conducted by pharmacists (123, 128, 130, 135). Nurses conducted medication reviews within two days after admission in one interventional study (131).

In a prospective, non-randomized controlled trial, two clinical pharmacists completed medication reconciliation at admission and conducted medication reviews weekly during hospitalization, at discharge and three months after discharge to identify and collaboratively resolve DRPs with physicians (123). The reviews at admission, discharge and follow-up included a comprehensive patient interview and the assessment of drug history and ADEs. The medication charts were checked for interactions with two DDI programs. Furthermore, the pharmacists participated in multidisciplinary ward rounds (six times per week). 63-87 DRPs per 1000 patient-days or 3.0 ± 2.7 DRPs per patient were identified at admission (123). In the intervention group, 5.8 DRPs per 1000 patient days or 0.4 ± 0.9 DRPs per patient remained unsolved compared to 76.9 per 1000 patient days or 2.3 ± 2.1 per patient in the control group (123), which was a significant reduction by -1.8 DRP per patient (95% CI: 1.5–2.1, p<0.001) (123). 88.6% of pharmaceutical recommendations were accepted by ward staff (123). As a further method, the Medication Appropriateness Index (MAI) score (137) was measured and significantly reduced at discharge and follow up in the intervention group (p<0.001) by -1.4 points per patient (95% CI: 0.8–2.1) and -1.2 points (95% CI: 0.6–1.9), respectively (123), indicating an increase in drug treatment appropriateness.

A second prospective, interventional before-and-after study assessed DRP identification by a clinical pharmacist through medication review using patients’ full records and pharmaceutical interventions to solve them (130). DRPs were documented and proposed interventions communicated via forms to physicians, and the pharmacist visited physicians to resolve questions before physicians decided whether to accept recommendations. 60% of DRPs with known outcome remained unsolved as 34% of these were only partially solved and 66% did not have to be or could not be solved (130). The number of prescribed drugs per patient was significantly reduced from 4.2 to 3.4 after the intervention (p=0.002) (130). Intervention acceptance by physicians was not specifically reported.

A different approach to identify and solve potentially inappropriate prescriptions (PIP) at admission was used in a controlled, interventional before-and-after study during which registered nurses conducted medication reviews within two days after admission, compared to usual care (131). Nurses participating in the interventional arm received an educational course on general pharmacology, psychopharmacology and treatment principles for some of the major mental disorders, principles for medication reviews, identification and documentation of PIP. In both groups, senior clinical pharmacology physicians recorded the number of PIP per patient, serving as a gold standard. The number of PIP per patient and the number of patients with at least one PIP were not significantly changed by the intervention (p=0.59 and p=0.14, respectively). During the intervention, PIPs were altered by physicians in response to nurses’ recommendations in 34% (131). Of the PIPs identified by the nurses and consequently changed by the physicians, only 17% were also PIPs identified and assessed for severity by the senior clinical pharmacology physicians (131). While senior clinical pharmacology physicians identified PIPs in 29.6% of prescriptions also assessed by nurses, nurses only identified 17% of these (131).

Grasso et al. (135) focused on transcription errors in discharge medication lists on hand written transcriptions compared to discharge lists generated by personal digital assistants (PDA). Significantly fewer PDA-generated discharge lists contained errors (8%) compared to manual transcriptions (22%, p<0.05) (135).

DDISs were retrospectively assessed by a clinical pharmacist before and after two educational classes on DDIs by a clinical pharmacist and implementation of a pharmacist consultation service consisting of DDI checks based on chart reviews (134). Significantly less DDIs per patient were identified after the intervention (2.8 vs. 3.4, p=0.04) with an overall reduction of potential DDIs by 44%. Clinically relevant DDIs were reduced by 78% through pharmacist interventions (134). The intervention acceptance rate was not reported.

An interdisciplinary concept to optimize patient safety including one group with a pharmacist intervention and a second group with a psychologist intervention was tested in a small pilot study (128). In the pharmacist’s group, DDI checks were performed by a pharmacist using two databases, clinically relevant DDIs were communicated to physicians in written form and pharmaceutical counseling (60 minutes/patient) on drug effects and side effects was offered to patients where deemed reasonable. If the pharmacist recommended prescription changes, they were discussed with a physician prior to patient counselling. In the psychologist’s group, psychological counseling (60 minutes/patient) was offered to patients focusing on the necessity of drug treatment, increasing expectancy of drug efficacy, decreasing anxiety and fears towards intake of antidepressants (128). While expectedly, the number of DDIs per patient was not changed in the psychologist’s group, it was significantly decreased by 38.9-77.8%, depending on the DDI database used from 9 (median: 0.6 DDIs per patient) to 3 DDIs (German DDI database) or 36 (median: 2.7 DDIs per patient) to 22 DDIs (American DDI database)) (p<0.05) (128). Medication adherence was measured using the Medication Adherence Rating Scale (MARS) score. No significant differences were found between groups in MARS-score before and after the interventions but there was a significant effect over time (p<0.05) (128).

#### 3.4.2 Interventions to manage ADRs

In an early study including psychiatric patients between 1996 and 1997, clinical pharmacy services were assessed in a psychiatric inpatient setting in the USA (125). Services included attending morning report and staff briefings, performing baseline assessments and weekly reviews, providing pharmacotherapy recommendations, obtaining medication histories, reviewing drug administration records daily, monitoring for ADRs, conducting weekly medication education classes, and counseling patients before discharge. Significant differences were seen on the Abnormal Involuntary Movement Scale (AIMS) (mean ± SD % change in score: 3.5 ± 12.1, p=0.024), the Barnes Akathisia Rating Scale (BARS) (27.0 ± 52.1, p=0.042), and the Simpson-Angus Rating Scale for Drug-Induced Extrapyramidal Symptoms (21.9 ± 31.9, p=0.002), with more pronounced positive change observed in the experimental group (125). 94% of pharmaceutical recommendations were accepted by the treating physicians (125).

Another interventional study prospectively assessed ADRs in patients who received divalproex sodium with a slow titration or loading regimen and did not find statistically significant differences between groups (2.2 ADRs per patient in both groups), suggesting, that slow titration does not increase the tolerability of divalproex sodium (126).

#### 3.4.3 Interventions to improve medication adherence

Three articles reported results of interventions to improve patient compliance to their drug treatment. Two focused on patients with schizophrenic disorders in a psychiatric hospital (133) and in a department of forensic psychiatry (129) and one study included patients with different psychiatric diagnoses such as schizophrenia, bipolar depression, post-traumatic stress disorder, and was conducted at an acute day care program (127).

The role of a clinical pharmacist in improving compliance in schizophrenic patients was studied in a prospective interventional study in Turkey during which patients received an educational brochure and an oral training by a clinical pharmacist at discharge (133). After the intervention, 17.5% of patients and therefore 70% less patients showed poor compliance compared to 57.5% before the educational intervention (133). There was a statistically significant increase in compliance as quantitatively assessed by the MARS after drug education (6.60 ± 2.23 to 8.60 ± 1.29, p<0.001) (133). Furthermore, Yalcin et al. determined the total BARS score and therefore the presence of ADRs as the most important impact factor for compliance (p=0.012) (133).

In the second study, a medication discontinuation program by a multidisciplinary team led to a significant increase in Drug Attitude Inventory (DAI-30) (p=0.002) from -2.6 ± 13.2 before to 18.3 ± 9.2 after the intervention and to 19.9 ± 8.5 in the recovery phase (129) and therefore to an increase in treatment adherence in forensic patients with schizophrenia. The program included psychoeducation of the patients, followed by a medication discontinuation period using monitoring sheets every day to confirm warning signs for readministration criteria, and a medication readministration period.

In the third intervention to improve compliance, microelectronic devices and feedback sessions with patients were used to monitor the extent to which patients took their drugs as prescribed (127). Medication bottle caps with digital displays showing the number of times the bottle had been opened that day, and number of hours since the previous opening were distributed to the patients in the intervention group. Patients were taught how to develop cues fitting into their lifestyle to remember dose times. They were also instructed to regularly check the display on the medication bottle cap to see when the next dose was due (127). In the intervention group, a significant improvement of compliance was achieved with mean one-month compliance rates before the first feedback session of 81 ± 22% compared to 68 ± 27% in the control group (p=0.023). The mean overall compliance was also significantly higher in the intervention group with 76 ± 22% compared to 57 ± 30% in controls (p=0.008) (127).

#### 3.4.4 Implementation of dispensing aid systems to prevent medication administration errors

Two different dispensing aid systems were studied with regard to the prevalence of MAEs (124, 132).

MAEs were identified through direct observation of 60 ward rounds by a pharmacist for three weeks each before and after an automated dispensing cabinet was installed at a psychiatric ward (124). The MAE rate was non-significantly reduced from 8.9% to 7.2% of administered medications (p=0.065) (124). Furthermore, the reduction in error rate was not clinically significant as only errors without any potential of harming a patient were reduced (124). Errors with the potential for harm were not reduced (124). However, the mean time necessary for nurses to administer a dose of medication was significantly decreased from 2.94 min to 2.37 min, thus 0.57 min per dose were saved in nursing time (p=0.006, 95% CI 0.17 to 0.97 min). On the study ward, this would correspond to a total saving of about 66 min of nursing time per day (124).

A different approach was tested in Japan where incident reports issued by nurses, doctors, pharmacists, occupational therapists and medical clerks were studied for 18 months each before and after a medication cart equipped with a palm vein authentication system was introduced (132). The error rate due to misidentification of patients was significantly reduced from 0.17% to 0.06% of patients (p<0.0001) (132). Overall, MAEs were reduced from 0.20% to 0.14% of patients (132).

## 4 Discussion

### 4.1 What are the most frequent DRPs and DRP subtypes in adult psychiatric inpatients?

Among the included studies, PEs, and among these, DDIs were reported with the highest overall prevalence of DRP subtypes in adult psychiatric inpatients with a substantial share of QT-DDIs.

In line with this overall impression from the published literature, DDIs were reported as the most common DRP among the study subjects in several of the included articles (110). However, in a German study during which clinical pharmacists worked collaboratively with psychiatrists on psychiatric wards, provided pharmaceutical counseling regarding disease and drugs to patients and participated in multidisciplinary ward rounds six times per week, DDIs were detected more seldomly than other DRPs, such as a complex therapy regimen, no or inadequate TDM or insufficient or untreated indication (123). Some DRPs such as ADRs may be underreported in studies using chart and record review for DRP detection, as in this setting ADRs are only identifiable if they were documented by the ward staff. Furthermore, problems with drug or dose selection and correspondingly, inadequate TDM, can be identified more easily if the responsible professional knows the patient including his or her diseases, attitudes towards and difficulties with drug treatment and his or her clinical presentation. Therefore, it is possible that studies using chart and record reviews as the only DRP detection method underestimate the prevalence of DRPs which are not formal PEs or content-related PIPs.

### 4.2 Which interventions have been tested to solve DRPs in adult psychiatric inpatients?

Clinical interventions studied in psychiatric inpatients included clinical pharmacy services on the ward, educational classes by pharmacists, psychologists or a multidisciplinary team, medication review with and without patient interviews by pharmacists or nurses, and the implementation of digital tools such as dispensing cabinets and prescribing tools.

A recently published German study retrospectively assessed the prevalence of DRPs before and after pharmacist-supported CPOE implementation in psychiatric inpatients and found a significant reduction by almost 50% after CPOE implementation, mainly through the prevention of PEs (136). In this study, DDIs, including DDIs between potentially QT-prolonging drugs, were frequent DRPs both before and after the intervention consisting of CPOE implementation with an integrated clinical decision support system and regular plausibility checks by clinical pharmacists (136). However, there was a tendency towards fewer DDIs after CPOE-implementation and pharmaceutical validation of the prescribed drugs. Compared to another German study included in this review where a more patient-centered approach was used (123), more DRPs remained unsolved at discharge (136).

Implementation rates of interventions differed between studies in which professionals with different backgrounds gave clinical recommendations. In a Danish study on the role of nurses in identification and solution of PIPs, only 34% of prescriptions were altered or written by physicians following the nurses’ suggestions and of these altered prescriptions, only 17% were also PIPs identified and assessed for severity by senior clinical pharmacology physicians (131). In contrast, 88.6% to 94% of pharmaceutical recommendations by clinical pharmacists working on psychiatric wards were accepted by ward staff (123) including treating physicians (125). These findings are supported by evidence from a study on outcomes of medication reconciliation in cardiology when performed by clinical pharmacists compared to nurses which reported that pharmacists spent significantly less time on medication reconciliation and physicians agreed significantly more often with pharmacists on clinical relevance (138).

Nonetheless, not all DRPs identified by clinical pharmacists are necessarily clinically relevant. Following clinical pharmacists’ written recommendations after chart and patient record review, 60% of DRPs with known outcome remained unsolved but 66% of these unsolved DRPs did not have to be or could not be solved (130). Likewise, in a recently published study, only 54% of clinical pharmacists’ interventions in medication charts after simple medication chart reviews were fully implemented by the treating physicians (136).

As one conclusion from the heterogenous articles included in this review and in line with results from a retrospective study on clinical pharmacist interventions during interdisciplinary rounding in Slovenia (139), it is possible that pharmaceutical recommendations regarding medication treatment of psychiatric inpatients gain clinical relevance and therefore achieve better acceptance by physicians if the clinical pharmacist works on the ward as part of a multidisciplinary team. Recommendations made without actually knowing the individual patient are more superficial and not tailored to the patient’s individual needs.

Furthermore, it is possible that psychiatric-specific training and experience of the pharmacists may improve their quality of care for this clientele. For example, the Board-Certified Psychiatric Pharmacist (BCPP) credential was established in the USA as a Board of Pharmacy Specialties certification, demonstrating that pharmacists are able to manage psychiatric disorders after appropriate training (140). A very recently published systematic literature review of the impact of psychiatric pharmacists included 202 primary literature articles highlighting the impact of psychiatric pharmacists on patient-level outcomes published between 1961 and 2022 (140), including 36 studies from inpatient settings. Overall, the review authors identified response to study treatment as the most common outcome measure in 141 total studies (69.5%), among other outcome measures such as medication-based, patient experience and adherence, adverse outcomes and cost-based outcomes (140). They did not explicitly assess the prevalence or solution of DRPs as an outcome measure but reported that most of the diverse outcomes showed positive results (140). Another review outlined the role of psychiatric pharmacists in improving patient outcomes both in inpatient and outpatient settings (141).

Although it is difficult to compare the interventions directly, some learnings may be summarized from the reported data:

- A well-structured medication reconciliation process including patient interviews is the first step towards identification of DRPs and therefore a successful medication management. If unintended medication discrepancies are not resolved at admission, they might lead to MEs with the potential for ADRs through hospitalization and discharge to ambulatory care.
- Clinical pharmacists were involved in 7 of the 13 included interventional studies and were able to identify DRPs and recommend clinical interventions from admission to discharge of psychiatric inpatients.
- Clinical pharmacists identified more medication discrepancies than nurses (65), potentially due to the advanced knowledge on pharmacotherapy like on drug formulations.
- During hospitalization, an interprofessional collaboration and integration of clinical pharmacist services on the ward helps to identify clinically relevant DRPs, such as complicated therapy regimens or missing TDM in cases of ADRs or insufficient treatment effectiveness.
- Furthermore, clinical pharmacists may support psychologists in interventions to improve medication compliance.
- An optimal medication management for psychiatric inpatients continues throughout discharge with the transition of the patient to ambulatory care. Again, clinical pharmacists are able to identify unintended medication discrepancies at discharge, discuss them with the treating psychiatrists and resolve problems before the patient leaves the psychiatric department.
- The medication management process should be supported by digital prescribing support tools, such as CPOE systems with integrated clinical decision support to reduce PEs.

### 4.3 Limitations

There are several limitations to this systematic review that need to be addressed. A systematic approach was used to identify the reported prevalence rates of different DRP categories among the published literature. However, the methods used for detection and classification of DRPs and the reporting qualities of included articles were heterogenous, the mean sample sizes were rather small, many studies did not calculate an explicit denominator to ensure comparability of the reported rates, and many studies failed to apply validity or reliability measures. In order to facilitate the interpretation of the different included results, a denominator (DRPs per 1000 patient days or DRPs as percentage rate of all prescriptions or patients) was calculated by the review’s authors for all studies reporting sufficient data. Mann et al. (143) previously discussed the methodological issues regarding the detection and classification of MEs and the methods that were used in other fields of medicine at the time of publication (2008) with an emphasis on their potential application to psychiatric care.

Furthermore, different definitions of what constitutes a DRP or a DRP category influence the reported DRP rates. For this reason, PIMs were excluded from this review as the methods used for their identification differ enormously among studies and their clinical relevance depends on the individual patient and his or her risk factors for ADRs under therapy with a PIM.

The rate of DRPs identified in the included studies differed depending on the methods used for their detection, on the profession of the study investigators, data collectors or professionals conducting the interventions and on the focus of investigated DRPs. Jayaram et al. calculated a ratio of self-reported errors per 1000 patient days to audited errors per 1000 patient days of 1:21.28 in 2005 and 1:23.64 in 2007 (78). Correspondingly, the reported DRP rates were much lower in studies where incident reports were used as the sole method to detect DRPs. On the other hand, in prospective studies or in studies which used a mixed-methods approach of direct patient observations and chart reviews, e.g. for ADRs (100), considerably higher prevalence rates of DRPs, e.g. of PEs, including formal errors (79), were identified than in retrospective chart reviews.

A limitation of the methods used in data analysis of this review was that the DRP rate was calculated as a percentage of patients by dividing the number of DRPs by the number of patients observed in the respective study multiplied by 100. However, some studies reported only the percentage of patients who experienced at least one DRP. Those patients could have experienced more than one DRP, e.g. ADR, thus, if the total number of ADRs would have been reported, the percentage of patients with an ADR would have been higher. Sander et al. (61) reported that 55% of patients on the gerontopsychiatric ward experienced at least one ADR, whereas 37% of patients in clinical social psychiatry presented with at least one ADR. However, the total number of 102 ADRs in 88 patients (or 208 cases) resulted in 1.2 ADRs per patient (or 0.5 per case), which would be equivalent to 116% of patients (or 49% of cases). Therefore, the percentage rates of DRPs cannot always be compared directly between studies, depending on the presentation of study results.

Overall, the evidence of prevalence data included in this review was rated as low to very low according to GRADE. However, there is no formal guidance from GRADE for systematic reviews of prevalence and cumulative incidence, but grading the overall prognosis or baseline risk is acceptable (144). We chose to apply the GRADE criteria to the evidence summarized in this review to objectively point out methodological issues in many studies reporting prevalence data of DRPs in psychiatric inpatients.

There were some deviations from the original study protocol which were pointed out in the respective methods sections. They were added after initial registration of the study protocol to increase reproducibility of the search and selection process of this review.

Furthermore, due to the nature of this review, as many as 88 articles were included in data synthesis which limited the details presented on each single included article. However, it is possible for clinicians and researchers to explore more details of the included studies in the interactive **Figures 2**, **3** and **5**. Therefore, using the results of this review, it is possible to inform future clinical decisions on interventions to optimize the drug treatment process in psychiatric inpatients.

Despite the limitations of this review including the heterogenous evidence from the included studies and the review methods used, an up-to-date overview of the existing literature was created on the prevalence of DRPs and interventions to solve or prevent them in psychiatric inpatients. In order to inform health professionals of the most important results of this systematic review, interactive visualizations were created using flourish.studio, as recommended in a recent review on data visualizations in scoping reviews (145).

### 4.4 Recommendations for policy and practice

Only seven of the included 88 articles reported the use of TDM with regard to DRPs in psychiatric inpatients. TDM might still not be used frequently enough in psychiatric practice although its benefits towards prevention of adverse clinical consequences have been studied extensively, such as inadequate treatment efficacy due to plasma concentrations below or ADRs due to concentrations above the therapeutic reference range, especially caused by pkDDIs (26, 146). Furthermore, it has been reported recently that patients who received TDM measurements of antidepressants at admission had a significantly shorter length of hospital stay compared to patients whose drug blood concentrations were measured later during hospitalization (147). Apart from the individual patient’s treatment effectiveness and safety, shorter lengths of stay may generate savings which exceed the costs for TDM, e.g. for citalopram (148). Both physicians and clinical pharmacists working on psychiatric wards should become familiar with TDM guidelines (26) to use TDM measurements correctly and in the recommended drugs.

In addition, the beneficial outcomes of pharmacists serving as independent prescribers have been described for patients with mental diseases in primary care, e.g. in Scotland (142). None of the studies included in this review assessed the impact of clinical pharmacists as independent prescribers in mental health hospitals. However, there is evidence that pharmacists make fewer prescribing errors than physicians (149). Therefore, future research should assess clinical pharmacists as independent prescribers in psychiatric care.

Following the World Health Organization’s recommendation to provide patients on polypharmacy with medication reviews to reduce drug-related harm, the German legislation was amended in 2020 to entitle patients taking five or more drugs for at least the following 28 days in community care to a medication review by community pharmacists (150, 151). Community pharmacists get reimbursed by the German health insurance companies for these medication reviews consisting of a brown-bag-review and a systematic check for DRPs. To date, there is no equivalent reimbursement strategy for medication reviews conducted by clinical pharmacists in hospitalized patients in Germany.

Therefore, in line with the position paper by the European Society of Clinical Pharmacy Special Interest Group on Mental Health (152) we recommend that legal reimbursement strategies for the conduct of clinical pharmacy services on psychiatric hospital wards will be discussed on national levels, where not implemented yet. Additionally, professional associations such as the German Association of Hospital Pharmacists should develop special courses for psychiatric pharmacists to ensure the highest possible quality of psychopharmacologic treatments in psychiatric clinics.

For clinical practice, we recommend that psychiatric hospitals establish clinical pharmacy services on the wards including medication reconciliation, educational classes, medication reviews, interdisciplinary ward rounds, and patient counseling. This may be supported by digital tools for clinical decision-making.

Furthermore, researchers should include comparable clinical outcome measures and if possible, prospective randomized controlled designs when planning future interventional studies.

### 4.5 Conclusions

The most frequent DRPs in psychiatric inpatients are related to prescribing errors and drug interactions. Clinical pharmacists can identify and solve DRPs in psychiatric patients. The evidence summarized in this systematic review supports the establishment of hospital pharmacists on psychiatric wards.

## Data Availability

The original contributions presented in the study are included in the article/**Supplementary Material**. Further inquiries can be directed to the corresponding author/s.
