# Supplementary material for: Prevalence and solving strategies of drug-related problems in adult psychiatric inpatients - a systematic review
Source: Front Psychiatry. 2024 Dec 4;15:1460098. doi: 10.3389/fpsyt.2024.1460098 (PMC11652846; doi:10.3389/fpsyt.2024.1460098)
Supplement: Supplementary file 1 [file DataSheet1.pdf]

# *Supplementary Material*

## 1 Search strategies

The following search strategies were used for the systematic literature search.

### 1.1 PubMed:

((("drug related problems"[Title/Abstract] OR "adverse drug events"[Title/Abstract] OR "medication errors"[Title/Abstract] OR "adverse drug reactions"[Title/Abstract] OR "drug interactions"[Title/Abstract] OR "contraindications"[Title/Abstract] OR "combination"[Title/Abstract]) AND ("psychiatry"[Title/Abstract] OR "mental health"[Title/Abstract]) AND ("inpatients"[Title/Abstract] OR "hospital"[Title/Abstract] OR "tertiary care"[Title/Abstract] OR "day hospital"[Title/Abstract])) NOT ("pediatric"[Title/Abstract] OR "children"[Title/Abstract] OR "adolescent"[Title/Abstract])) AND (1999:2022[pdat])

### 1.2 Scopus:

( ( ( TITLE-ABS-KEY ( "drug related problem*" ) OR TITLE-ABS-KEY ( "adverse drug event*" ) OR TITLE-ABS-KEY ( "adverse drug reaction*" ) OR TITLE-ABS-KEY ( "medication error*" ) OR TITLE-ABS-KEY ( "drug interaction*" ) OR TITLE-ABS-KEY ( "contraindication*" ) OR TITLE-ABS-KEY ( combination ) ) ) AND ( ( TITLE-ABS-KEY ( psychiatry ) OR TITLE-ABS-KEY ( "mental health" ) ) ) AND ( ( TITLE-ABS-KEY ( inpatients ) OR TITLE-ABS-KEY ( hospital ) OR TITLE-ABS-KEY ( {tertiary care} ) OR TITLE-ABS-KEY ( {day hospital} ) ) ) ) AND NOT ( ( TITLE-ABS-KEY ( pediatric ) OR TITLE-ABS-KEY ( children ) OR TITLE-ABS-KEY ( adolescent ) ) ) AND ( LIMIT-TO ( PUBYEAR , 2022 ) OR LIMIT-TO ( PUBYEAR , 2021 ) OR LIMIT-TO ( PUBYEAR , 2020 ) OR LIMIT-TO ( PUBYEAR , 2019 ) OR LIMIT-TO ( PUBYEAR , 2018 ) OR LIMIT-TO ( PUBYEAR , 2017 ) OR LIMIT-TO ( PUBYEAR , 2016 ) OR LIMIT-TO ( PUBYEAR , 2015 ) OR LIMIT-TO ( PUBYEAR , 2014 ) OR LIMIT-TO ( PUBYEAR , 2013 ) OR LIMIT-TO ( PUBYEAR , 2012 ) OR LIMIT-TO ( PUBYEAR , 2011 ) OR LIMIT-TO ( PUBYEAR , 2010 ) OR LIMIT-TO ( PUBYEAR , 2009 ) OR LIMIT-TO ( PUBYEAR , 2008 ) OR LIMIT-TO ( PUBYEAR , 2007 ) OR LIMIT-TO ( PUBYEAR , 2006 ) OR LIMIT-TO ( PUBYEAR , 2005 ) OR LIMIT-TO ( PUBYEAR , 2004 ) OR LIMIT-TO ( PUBYEAR , 2003 ) OR LIMIT-TO ( PUBYEAR , 2002 ) OR LIMIT-TO ( PUBYEAR , 2001 ) OR LIMIT-TO ( PUBYEAR , 2000 ) OR LIMIT-TO ( PUBYEAR , 1999 ) ) AND ( LIMIT-TO ( LANGUAGE , "English" ) OR LIMIT-TO ( LANGUAGE , "German" ) OR LIMIT-TO ( LANGUAGE , "French" ) )

### 1.3 Google Scholar:

allintitle: mental illness adverse OR event OR reaction OR medication OR error OR interaction OR contraindication -ambulant -pediatric -children -adolescent

allintitle: psychiatry adverse OR event OR reaction OR medication OR error OR interaction OR contraindication -ambulant -pediatric -children -adolescent

psychiatry OR "mental health" "drug related problems" -pediatric -OR -children -OR -adolescent -OR -kinder -OR -pädiatrie -OR -jugendliche

allintitle:("drug related problems"|"adverse drug events"|"medication errors"|"adverse drug reactions"|"drug interactions"|contraindications|combination) AND (psychiatry OR "mental health") AND (inpatient OR hospital OR "tertiary care" OR "day hospital")

### 1.4 ClinicalTrials.gov

Condition or disease: Mental Illness

Other terms: drug related problems

### 1.5 Cochrane Library/Cochrane Database of Systematic Reviews

ID Search Terms

#1 ("drug related problem"):ti,ab,kw OR ("adverse drug event"):ti,ab,kw OR ("adverse drug reaction"):ti,ab,kw OR ("contraindication"):ti,ab,kw OR ("drug interaction"):ti,ab,kw (Word variations have been searched)

#2 ("psychiatric"):ti,ab,kw OR (mental health):ti,ab,kw (Word variations have been searched)

#3 ("inpatient"):ti,ab,kw OR (hospital):ti,ab,kw OR ("tertiary care"):ti,ab,kw OR ("day hospital"):ti,ab,kw (Word variations have been searched)

#4 ("pediatric"):ti,ab,kw OR (children):ti,ab,kw OR (adolescent):ti,ab,kw (Word variations have been searched)

#5 #1 AND #2 AND #3

#6 #5 NOT #4 (Word variations have been searched)

## 2 Supplementary Figures and Tables

**Table 1:** List of excluded studies assessed for eligibility by full text screening and the respective justifications for exclusion is uploaded separately as an Excel data sheet.

**Table 2:** Data extraction form of included studies, including results of quality assessments is uploaded separately as an Excel data sheet.
